# Supplementary material for: Chromosome-level genome and population genomics reveal evolutionary characteristics and conservation status of Chinese indigenous geese
Source: Commun Biol. 2022 Nov 7;5:1191. doi: 10.1038/s42003-022-04125-x (PMC9640629; doi:10.1038/s42003-022-04125-x)
Supplement: Supplementary file 2 — Supplementary Information [file 42003_2022_4125_MOESM2_ESM.pdf]

Chromosome-level genome and population genomics reveal evolutionary characteristics and conservation status of Chinese indigenous geese

Jing Ouyang[1†], Sumei Zheng[1,2†], Min Huang[2], Hongbo Tang[1], Xiaohui Qiu[3], Shoujin Chen[4], Zhangzhang Wang[4], Zhongdong Zhou[4], Yuren Gao[1], Yanpeng Xiong[1], Guohua Zeng[3], Jimin Huang[3], Jiugen He[3], Jun Ren[2], Hao Chen[1*], Xueming Yan[1*]

Supplementary Methods

Supplementary Method 1. Genome assembly

Stage 1: PacBio assembly

Genomic DNA was isolated from a female Xingguo gray goose (XGG) through a traditional phenol-chloroform protocol, the qualified genomic DNA was fragmented by 26 G Needle, and then PacBio SMRTbell libraries (20 Kb insert) were prepared for single-molecule real-time sequencing with a sequencing depth of 101.33× and raw data was 123.62 Gb (Supplementary Figure 1 and Supplementary Table 1).

Using wtdbg v2.4[1] for *de novo* assembly based on the data generated by PacBio sequencing above, the assembly process was as follows:

(1) Alignment: KBM (Kmer-Bin-Mapping) algorithm was used to cut the sequenced long reads into several bins with 256-bp as a unit without overlapping. The k-mers in the fragment were then classified into a bin, and a k-mer has at least 4 × 256-bp. If most k-mers in a bin occur at a higher frequency, the bin will be filtered out as a high repeat bin. Finally, the linear relationship between matched bins in the sequence was searched by dynamic programming.

(2) Assembly: FBG (Fuzzy Bruijn Graph) was used to break reads into multiple 1024-bp k-mers as vertices in FBG graph, and then these points were connected to form FBG graph according to the reading path, and contigs were obtained by simplifying FBG graph.

(3) Polish: To improve the assembly quality, the reads generated by PacBio sequencing

were used to polish the assembled contigs. The high-confidence bases were obtained by comparing and correcting each other between sequences, and finally high-quality consensus sequences were produced.

Stage 2: Super-scaffolding

10× Genomics is based on GemCode platform to amplify the long fragment sequence, introduces barcode sequence and adapter primers, then break the sequences into appropriate fragments for sequencing, and assemble multiple reads through barcode sequence information to obtain 99.10× Linked-reads data (120.90 Gb; Supplementary Figure 1 and Supplementary Table 1).

The Linked-reads generated by the 10× Genomics were assembled with the consensus sequences of Stage 1 using fragScaff v140324.1[2] to generate the supper-scaffold, and the final 10× Genomics assembly version was obtained.

Stage 3: Super-scaffold gap filling

To improve the assembly of complex regions of the genome, we constructed the BioNano library for high-depth sequencing (137.47×, 167.72 Gb) using the Irys system, and used the obtained physical map to span repetitive fragments and some regions containing complex elements to acquire highly continuous Super-scaffold (Supplementary Table 1).

The process of BioNano assisted genome assembly was as follows:

(1) The assembled version of Stage 2 was electronically digested and converted into a cmap file, which was recorded as NGS cmap.

(2) Enzyme site spacing was normalized by bionano data bnx, NGS map and estimated genome size, and the optimal assembly parameters were screened out. BioNano data were assembled to generate a BioNano assembly version, which was recorded as BNG cmap.

(3) The bnx, NGS map, BNG map and initial assembly version were based on hybridScaffold software[3], and reasonable parameters were set to obtain the final assembly version of BioNano.

Stage 4: Assembly polishing using Illumina reads

In addition, we sequenced by constructing a 350-bp insert library based on a standardized process of Illumina NOVASEQ 6000 platform with a sequencing depth of 111.59×, resulting

in a total of 136.14 Gb of data (Supplementary Table 1). Using Illumina pair-end reads to polish the assembled version of Stage 3 and the specific process was as follows:

(1) The reads generated by Illumina sequencing were aligned to the assembled version of Stage 3 by BWA v0.7.17[4], and the Sequence Alignment/MAP format (BAM) files were obtained and sorted.

(2) Subsequently, combined with the mapping results, the assembled version was polished iteratively 2 to 3 times to improve the single-base correct rate using Pilon v1.23[5].

Stage 5: Hi-C aided chromosome-level whole-genome assembly

Hi-C technology obtains the interaction information of spatially connected DNA fragments through special experimental techniques. Different contigs or scaffolds are classified into different chromosomes according to the probability of internal chromosome interaction significantly higher than that between chromosomes. Contigs or scaffolds of the same chromosome are ordered and orientated according to the probability which decreases with the increase of the interaction distance. To anchor assembled scaffolds onto the chromosome, we constructed the Hi-C library and obtained sequencing data (112.91×, 137.75 Gb) via the Illumina HiSeq X Ten platform. The detailed auxiliary anchoring process was as follows:

(1) Alignment: Hi-C data were aligned to the assembled scaffolds constructed by Stage 4 through BWA, and duplicated and non-aligned data were removed by SAMtools v1.10[6] to obtain high-quality alignment data while extracting reads near the restriction site for auxiliary assembly.

(2) Clustering: Through the physical coverage information of the comparison results, the incorrect assembly from the previous step was interrupted and corrected. If read pairs captured by Hi-C technology on two contigs, it is determined that there is an interaction between the two contigs. The greater the number of interacting reads on two contigs, the stronger the interaction, and the more they tend to cluster together. Subsequently, we counted the number of interactions between contigs to cluster the contigs and divided them into designated classes according to the number of chromosomes of the species.

(3) Sorting and orientation: According to the results of clustering, the position of each two contigs and the intensity of interaction, LACHESIS v201701[7]

(https://github.com/shendurelab/LACHESIS) was used to sort and orientate the assembled sequences to the chromosome level.

Finally, we performed artificial correction of the LACHESIS-assembled results and gap filling or sequence de-duplication to increase the accuracy and completeness of the assembled genome.

Assembly quality assessment

(1) Sequence consistency assessment

To evaluate the accuracy of genome assembly, we used BWA to re-align the Illumina pair-end reads to the assembled genome of XGG and counted the mapping rate, the coverage and sequencing depth of the genome to assess consistency of assembly and the homogeneity of sequencing. The alignment rate was about 99.19%, and the coverage rate was about 97.96%, indicating that the reads and the assembled genome had a good consistency (Supplementary Table 6). The heterozygous SNP ratio of XGG was 0.2777% and the homozygous SNP ratio was 0.0005%, which generally reflected the accuracy of genome assembly. This result indicated that the XGG genome had a higher single-base accuracy.

(2) Sequence integrity assessment

To further investigate the quality of the genome assembly, the assembled transcriptome Unigenes were mapped to the genome sequences and performed CEGMA v2.5[8] (Core Eukaryotic Genes Mapping Approach: http://korflab.ucdavis.eGO/dataseda/cegma/) and BUSCO v.5.2.2[9] (Benchmarking Universal Single-Copy Orthologs: http://busco.ezlab.org/) assessments. CEGMA assessment was to select conserved genes (248 genes) in 6 eukaryotic model organisms to form the core gene library, and combined with tblastn[10] and GeneWise[11] to evaluate the completeness of assembled genome. BUSCO assessment based on metaeuk v5.34[12] and hmmer v3.1[13] to assess the integrity of XGG genome assembly of 8,338 orthologous single-copy genes in the Aves database. Results showed that over 92.3% of 248 core genes could be covered by the genome indicating its completeness. BUSCO results also indicated that more than 95.7% of 8,338 avian orthologous genes could be found in the XGG genome, also reflecting its high quality (Supplementary Table 7).

Supplementary Method 2. Genome annotation

Stage 1: Repeat annotation

We used homologous alignment and *de novo* search to annotate whole-genome repeat sequences of XGG in our repeat annotation pipeline (Supplementary Figure 3). Tandem Repeat was extracted using Tandem Repeats Finder (TRF) v4.09[14] (http://tandem.bu.edu/trf/trf.html) by *ab initio* prediction. Based on the repeat sequence database RepBase[15] (http://www.girinst.org/repbase/), we used RepeatMasker v4.0.7[16] (http://www.repeatmasker.org/) software and its in-house scripts (RepeatProteinMask) with default parameters to identify and classify sequences similar to known repetitive sequences. And *ab initio* prediction built *de novo* repetitive elements database by LTR_FINDER v1.05[17] (http://tlife.fudan.edu.cn/ltr_finder/), RepeatScout v1.05[18] (http://www.repeatmasker.org/), RepeatModeler v1.0.3[19] (http://www.repeatmasker.org/RepeatModeler.html) with default parameters, then all repeat sequences with lengths >100bp and gap 'N' less than 5% constituted the raw transposable element (TE) library. A custom library (a combination of Repbase and our *de novo* TE library which was processed by uclust v1.2.22q[20] to yield a non-redundant library) was supplied to RepeatMasker for DNA-level repeat identification. Finally, we obtained 2.04% tandem repeats by TRF, 8.55% transposable elements by RepeatMasker and 4.55% transposable element proteins by RepeatProteinMask, and totally of 10.17% non-redundant repetitive sequences. Additionally, we annotated 0.16% DNA transposable elements, 6.26% LINE, 0.03% SINE and 2.54% LTR (Supplementary Tables 10–11).

Stage 2: Gene annotation

(1) Gene structure annotation

Structural annotation of XGG genome incorporates homology-based prediction, *de novo* prediction, and transcriptome data-based approach. First, the homology prediction was performed, using coding sequences of the homologous species (*Anas platyrhynchos*, *Anser cygnoides*, *Gallus gallus*, *Meleagris gallopavo*, and *Coturnix japonica*) to compare with the genome sequence of XGG through BLAST v2.28[10] (http://blast.ncbi.nlm.nih.gov/Blast.cgi), and then the matching proteins were aligned to the homologous genome sequences for

accurate spliced alignments with GeneWise v2.4.1[21] (http://www.ebi.ac.uk/~birney/wise2/) software which was used to predict gene structure contained in each protein region. Afterward, we conducted *de novo* prediction and used Augustus v3.3.3[22] (http://bioinf.uni-greifswald.de/augustus/), Geneid v1.4[23], Genescan v3.1.2[24], GlimmerHMM v3.04[25] (http://ccb.jhu.edu/software/glimmerhmm/) and SNAP v2013.11.29[26] (http://homepage.mac.com/iankorf/) to predict gene structure based on the statistical characteristics of Xingguo gray goose genome sequence data. To optimize the genome annotation, the transcriptome reads from different tissues that were aligned to XGG genome using TopHat v2.0.8[27] with default parameters to identify exons region and splice positions. The alignment results were then used as input for Cufflinks v2.1.1[28] with default parameters for genome-based transcript assembly. Furthermore, combining the genes supported by various methods through EVidenceModeler (EVM) v1.1.1[29] (http://evidencemodeler.sourceforge.net/) to generate a non-redundant and more complete gene set. In the end, using PASA v2.4.1[29] (http://pasa.sourceforge.net/) to correct the EVM annotation results, add UTR and variable shear, and other information to get the final gene set.

(2) Gene function annotation

After acquiring gene structure information, we further utilized Blastp v2.28[30] (E-value < 1e-5) to compare the sequences with protein databases such as SwissProt[31] (http://www.uniprot.org/), NCBI nr[32] (http://www.ncbi.nlm.nih.gov/protein), Pfam[33] (http://pfam.xfam.org/), KEGG[34] (http://www.genome.jp/kegg/) and InterPro[35] (https://www.ebi.ac.uk/interpro/) to predict the domain, the functions of encoding proteins of new genes, and the metabolic pathway and signal transduction pathway. Ultimately, we totally annotated 17,135 (98.2%) functional genes of 17,448 predicted genes, of which 16,381 (93.9%) genes from Swissprot database, 16,803 (96.3%) genes from NCBI nr database, 14,631 (83.9%) genes from KEGG database, 17,072 (97.8%) genes from InterPro database, 15,926 (91.3%) genes from GO[36] and 14,435 (82.7%) genes from Pfam database (Supplementary Figure 4 and Supplementary Table 9).

Stage 3: Annotation of ncRNAs

Annotations of noncoding RNAs include tRNAs, rRNAs, miRNAs, and snRNAs. The

tRNAscan-SE v1.4[37] (http://lowelab.ucsc.edu/tRNAscan-SE/) was used to identify the tRNA sequences in terms of the structural characteristics. According to the high conservation of rRNA, we used the rRNA sequences of the related species as reference to predict by BLAST. Moreover, the INFERNAL v1.1.2[38] (http://infernal.janelia.org/) in Rfam[39] was used to predict the sequence information of miRNA and snRNA on the genome through the covariance model of the Rfam family (Supplementary Table 12).

Supplementary Method 3. Identification of sex chromosomes

The sex chromosomes of XGG were determined based on the genomic sequence of avian chromosome-level genomes in the public database, which mainly encompasses four steps: segmentation of genomic sequence, homologous analysis of sequences, sequence-based screening and assessment. The specific process was as follows:

Step 1: Segmentation of genomic sequence

Firstly, we split sequences of XGG into short-reads with a size of 300 bp using in-house python script, and then filtered the reads with more than 10 ambiguous bases N. The remaining reads were used for subsequent alignment.

Step 2: Homologous analysis of sequences

We simultaneously downloaded the assembled sequences of 7 birds whose sex chromosomes have been identified (Supplementary Table 13). Then, we separately merged all Z and W sequences as the Z and W reference library and built the index files for each of them using BWA. The short-reads (300 bp) of XGG that split and filtered above were then mapped to the reference libraries and all the aligned reads were extracted by SAMtools.

Step 3: Sequence-based screening

Subsequently, we further carried out quality control on the aligned reads extracted above. We kept the reads with Mapping Quality > 30 by SAMtools and sorted the alignments according to scaffolds and read ID. Afterward, we filtered the alignments that were mapped on different sequences or different regions in one scaffold. Finally, the chromosome type of the scaffolds was inferred according to the proportion of aligned reads to the scaffold and

then calculated the cumulative length of the same chromosome type of scaffolds until the average length of the downloaded high-quality Z and W references.

Step 4: Sex chromosome assessment

Due to the close relationship between goose and duck, and considering the high assembly quality of Pekin duck, XGG and Pekin duck[40] were selected for collinearity analysis. We used the "One Step MCScanX-Super Fast" function of TBtools v1.06[41] to align the genome sequences of Pekin duck and XGG, merged the results of MCScanX to generate a collinearity file, and then visualized though the "File Transformat for MicroSynteny Viewer" function to further evaluate the sex-linked sequences. Next, with the re-sequenced mapping data of 162 Chinese domestic geese (Supplementary Table 15), the average sequencing depth of all sequences was computed using the 'samtool depth' command of SAMtools in 5-kb non-overlapping windows. We regarded the sequencing depth of autosomes as a normal value. The sequencing depths of Z and W chromosomes in females were approximately identical, and both were half of the autosomes. The average sequencing depth of Z chromosome in males was similar to that of autosomes, while the W chromosome was closer to zero. Based on the above identification results, we focused on Hic_3 and Hic_4 as Z and W chromosomes of XGG, respectively.

Supplementary Method 4. SNP calling

To improve the efficiency and accuracy of variants detection, we used the commercial software Sentieon v201711.03[42] on Tianhe-2 Supercomputer to identify SNPs and small Indels for 994 geese. First, we built the index file for XGG and aligned high-quality reads to the reference with BWA to generate Sequence Alignment/Map (SAM) files, which were then format transformed to BAM files and sorted by SAMtools. For the mapping results, "LocusCollectora" and "Realigner" functions from Sentieon were used to delete polymerase chain reaction (PCR) duplicates and calibrated according to the quality score. Next, "Haplotyper" function was applied to generate Genomic Variant Call Format (GVCF) files for each individual and separately performed joint calling for 772 XGG and 222 geese using "GVCFtyper" function. Finally, GATK v4.0.12[43]、BCFtools v1.15[44] and PLINK v1.9[45] were

used to filter SNPs with the following conditions:

(1) For 772 low-depth (1×) resequencing individuals: we call variants for 2 steps. For the first step, we retained SNPs with the parameters "QD (QualByDepth) < 2.0, FS (FisherStrand) > 60.0, MQ (RMSMappingQuality) < 40.0, SOR (StrandOddsRatio) > 3.0, MQRankSum (MappingQualityRankSumTest) < -12.5, ReadPosRankSum (ReadPosRankSumTest) < -8.0, SOR (StrandOddsRatio) > 3.0, MAF (Minor Allele Frequency) > 0.003, and call rate > 0.8". Secondly, we used SNPs obtained from the first step as a known data set to perform joint calling again. After filtering with the same conditions above, 163,067 SNPs were obtained to carry out whole genome imputation using STITCH v1.68[46] with the parameters of K = 40 and nGen = 50. Finally, a total of 12,415,004 SNPs was retained with MAF > 0.01 and call rate > 0.9.

(2) For 222 high-depth (10×) resequencing individuals: after joint calling, 13,008,900 SNPs were obtained after quality control with the parameters "QD < 2.0, FS > 60.0, MQ < 40.0, SOR > 3.0, MQRankSum < -12.5, ReadPosRankSum < -8.0, SOR>3.0, MAF > 0.05, and call rate > 0.9".

Supplementary Tables

Supplementary Table 1. Statistics of genome sequencing data of XGG.

Pair-end libraries	Insert size (bp)	Total data (Gb)	Read length (bp)	Sequence coverage (×)
Illumina reads	350	136.14	150	111.59
PacBio reads	20,000	123.62	15,280 (N50)	101.33
10× Genomics	600	120.90	150	99.10
Bionano	-	167.71	-	137.47
Hi-C	350	137.75	150	112.91
Total	-	686.12	-	562.4

Supplementary Table 2. Statistical results for each chromosome on XGG genome.

Hic_ID	Pseudo-chromosome	Cluster number	length (bp)	G+C (%)
Hic_0	1	7	209,937,520	40.16
Hic_1	2	7	161,260,155	40.04
Hic_2	3	5	121,836,162	39.94
Hic_3	38 (Z)	8	78,059,545	39.64
Hic_4	4 (W)	5	18,190,528	45.29
Hic_5	5	4	77,964,326	39.83
Hic_6	6	2	65,887,397	41.49
Hic_7	7	2	40,772,850	41.41
Hic_8	8	2	38,093,052	41.88
Hic_9	9	2	32,954,318	42.25
Hic_10	10	3	26,882,679	43.10
Hic_11	11	2	20,182,763	44.98
Hic_12	12	2	22,278,886	43.40
Hic_13	13	2	22,003,932	42.55
Hic_14	14	2	22,645,356	43.25
Hic_15	15	2	21,569,393	43.09
Hic_16	16	3	18,393,121	45.26
Hic_17	17	2	16,340,960	46.30
Hic_18	18	3	15,607,799	45.79
Hic_19	19	2	13,056,453	47.62
Hic_20	20	17	12,304,091	48.79
Hic_21	21	25	12,166,798	47.25
Hic_22	22	2	9,065,161	48.44
Hic_23	23	2	7,873,851	49.76
Hic_24	24	2	7,713,126	51.44
Hic_25	25	2	7,064,174	52.59
Hic_26	26	2	6,484,800	52.70
Hic_27	27	3	5,512,697	49.52
Hic_28	28	2	6,110,239	53.50
Hic_29	29	2	3,196,551	59.17
Hic_30	30	2	2,953,559	59.97
Hic_31	39	2	1,382,414	62.90
Hic_32	31	2	1,172,193	56.62
Hic_33	32	4	1,164,976	60.47
Hic_34	33	2	780,505	62.98
Hic_35	34	46	638,055	58.32
Hic_36	35	5	931,379	63.66
Hic_37	36	17	1,369,031	57.16
Hic_38	37	15	4,330,114	51.62
Scaffold *	-	2,203	27,335,139	-

Supplementary Table 3. The assembly rate of XGG.

**Number of anchored bases (bp)**	1,136,130,909
**Total number of bases (bp)**	1,163,486,048
**Anchored Rate (%)**	97.65

Supplementary Table 4. Nucleotide content of XGG.

Type	Number (bp)	% of genome
A	334,957,286	28.79%
T	335,072,751	28.80%
C	244,919,141	21.05%
G	244,557,032	21.02%
N	3,979,838	0.34%

Supplementary Table 5. Genome assemblies for scaffold N50 length comparison and BUSCO analyses.

Common name	Species	Assembly level	Assembly version
Xingguo gray goose	*Anser cygnoides*	Chromosome	This study
Tianfu goose	*Anser cygnoides*[47]	Chromosome	GCA_013030995.1
Zhedong white goose	*Anser cygnoides*[48]	Scaffold	GCF_000971095.1
Sichuan white goose	*Anser cygnoides*[49]	Scaffold	GCA_002166845.1
Bar-headed goose	*Anser indicus*[50]	Chromosome	GCA_006229135.1
Pink-footed goose	*Anser brachyrhynchus*[51]	Chromosome	GCA_002592135.1
African pygmy goose	*Nettapus auritus*	Chromosome	GCA_011076525.1
Canada goose	*Branta canadensis*	Chromosome	GCA_006130075.1
Chicken	*Gallus gallus*[52]	Chromosome	GCA_000002315.5
Turkey	*Meleagris gallopavo*[53]	Chromosome	GCF_000146605.3
Peregrine falcon	*Peregrine falcon*[54]	Chromosome	GCA_001887755.1
Bengalese finch	*Lonchura striata domestica*[55]	Chromosome	GCA_005870125.1
Pekin duck	*Anas platyrhynchos*[40]	Chromosome	GCA_003850225.1
Zebra finch	*Taeniopygia guttata*[54]	Chromosome	GCA_009859065.2
Kakapo	*Strigops habroptila*[56]	Chromosome	GCA_004027225.2
Superb fairywren	*Malurus cyaneus*[57]	Chromosome	GCA_009741485.1

Supplementary Table 6. Reads coverage of XGG.

Reads	Mapping rate (%)	99.19
**Genome**	Average sequencing depth	100.88
	Coverage (%)	97.96
	Coverage at least 4× (%)	97.87
	Coverage at least 10× (%)	97.76
	Coverage at least 20× (%)	97.56

Note: Coverage at least N×(%), the proportion of the genome covered with N× Reads.

Supplementary Table 7. The results of assembly quality assessment of CEGMA and BUSCO.

**CEGMA**		**BUSCO**		
complete	complete + partial	Complete	Fragmented	Missing
171 (68.95%)	230 (92.30%)	95.70%	1.0%	3.30%

Supplementary Table 8. Basic statistical results of gene structure prediction in XGG.

Method	Gene set	Gene number	Mean transcript length (bp)	Mean CDS length (bp)	Exons per transcript	Mean exon length (bp)	Mean intron length (bp)
De novo	Augustus[22]	20,204	14,520.68	1,313.99	7.75	169.54	1,956.45
	GlimmerHMM[25]	211,371	4,751.23	480.92	2.69	178.52	2,520.96
	SNAP[58]	66,746	25,705.76	663.00	5.52	120.04	5,536.67
	Geneid[23]	35,320	22,781.80	1,148.26	6.11	187.86	4,231.63
	Genscan[24]	43,982	19,943.00	1,368.08	7.97	171.71	2,666.07
**Homolog**	*Anser cygnoides*	33,702	9,941.82	959.90	4.96	193.37	2,265.80
	Anas platyrhynchos	21,983	15,062.37	1,263.90	6.67	189.36	2,431.61
	Coturnix japonica	19,020	17,262.92	1,405.10	7.64	183.90	2,388.01
	Gallus gallus	24,233	12,354.55	1,090.99	5.74	190.01	2,375.31
	Meleagris gallopavo	32,801	8,315.85	900.67	4.71	191.27	1,999.30
**RNA-seq**	PASA	60,697	14,925.87	1,132.57	6.88	164.57	2,344.99
	Cufflinks[59]	35,577	24,131.15	3,309.39	8.38	394.76	2,820.16
**EVM**		24,855	16,188.96	1,228.27	7.27	168.92	2,385.63
**Pasa-update***		24,688	17,192.64	1,244.96	7.34	169.66	2,516.29
**Final set****		17,448	23,019.79	1,533.17	9.52	160.98	2,520.65

Note: * includes the UTR region, others do not. ** contains the UTR region, which was obtained by removing the alternative splicing, selecting the longest transcript, removing redundant exons and filtering from the gene set of pasa2 update. Filtering conditions: overlap with TE greater than or equal to 20%, stop gain, only supported by *de novo* evidence, and rpkm expression in each tissue was less than 1.

Supplementary Table 9. Statistical results of gene function annotation.

Database	Gene number	Percent (%)
Swissprot	16,381	93.90
Nr	16,803	96.30
KEGG	14,631	83.90
InterPro	17,072	97.80
GO	15,926	91.30
Pfam	14,435	82.70
Annotated	17,135	98.20
Unannotated	313	1.80
Total	17,448	-

Supplementary Table 10. The predicted genome-wide repetitive sequences of XGG.

Method	Repeat Size (bp)	Percent (%)
TRF	23,771,262	2.04
RepeatMasker	99,737,512	8.55
RepeatProteinMask	53,067,289	4.55
Total	118,586,730	10.17

Supplementary Table 11. Whole genome transposable elements (TE) of XGG.

TE type	Denovo+Repbase Length (bp)	% in Genome	TE proteins Length (bp)	% in Genome	Combined TEs Length (bp)	% in Genome
DNA	1,737,587	0.15	218,250	0.02	1,919,410	0.16
LINE	67,441,314	5.78	45,338,969	3.89	72,973,327	6.26
SINE	292,458	0.03	0	0	292,458	0.03
LTR	28,477,183	2.44	7,597,143	0.65	29,561,807	2.54
Simple_repeat	2,171,633	0.19	0	0	2,171,633	0.19
Unknown	4,584,688	0.39	0	0	4,584,688	0.39
Total	99,737,512	8.55	53,067,289	4.55	103,475,240	8.87

Note: Denovo+Repbase is a library predicted by RepeatModeler, RepeatScout and LTR_FINDER software combined with RepBase nucleotide library, using Uclust software to integrate according to the 80-80-80 principle, and then using RepeatMasker software to identify TEs; TE proteins are based on RepBase protein library, using RepeatProteinMask software to identify TEs; Combined TEs is the result of integrating the above two methods and removing redundancy. Unknown indicates that the repeat sequence cannot be classified by RepeatMasker.

Supplementary Table 12. Noncoding RNA statistics in XGG.

ncRNA Type		Copy number	Mean length (bp)	Total length (bp)	% of genome
miRNA		424	85.59	36,290	0.003112
tRNA		371	74.68	27,706	0.002376
rRNA	rRNA	234	247.90	58,009	0.004975
	18S	20	683.45	13,669	0.001172
	28S	85	339.14	28,827	0.002472
	5.8S	8	155.38	1,243	0.000107
	5S	121	117.93	14,270	0.001224
snRNA	snRNA	346	126.76	43,858	0.003761
	CD-box	114	92.05	10,494	0.000900
	HACA-box	81	139.48	11,298	0.000969
	splicing	132	142.40	18,797	0.001612

Supplementary Table 13. Reference chromosomes used in sex chromosomes identification.

Chromosome	Species	Length (bp)	Assembly version
Z	*Anas platyrhynchos*[40]	84,547,829	GCF_015476345.1
Z	*Coturnix japonica*[60]	67,000,979	GCA_001577835.1
Z	*Gallus gallus*[52]	82,529,921	GCA_000002315.5
Z	*Numididae*[61]	75,261,281	GCA_002078875.2
Z	*Parus major*[62]	74,514,349	GCA_001522545.3
Z	*Taeniopygia guttata*[54]	78,980,481	GCA_008822125.1
W	*Anas platyrhynchos*[40]	16,693,329	GCF_015476345.1
W	*Coturnix japonica*[60]	122,254	GCA_001577835.1
W	*Gallus gallus*[52]	6,813,114	GCA_000002315.5
W	*Meleagris gallopavo*[53]	313,225	GCA_000146605.4

Supplementary Table 14. Sex-linked sequences identified in the genome of XGG.

**Chromosome type**	**Sequence ID**	**Length (bp)**
W	Hic_4	18,190,528
W	original_scaffold_2066_obj_pilon	6,053
Z	Hic_3	78,059,545
Z	fragScaff_scaffold_42_obj_pilon:::fragment_2:::debris	10,000
Z	Super-Scaffold_84_pilon:::fragment_4:::debris	10,000
Z	Super-Scaffold_29_pilon:::fragment_4:::debris	30,000
Z	original_scaffold_1774_obj_pilon	23,497
Z	original_scaffold_265_obj_pilon	31,285
Z	original_scaffold_1886_obj_pilon	14,535
Z	Super-Scaffold_66_pilon:::fragment_6:::debris	20,000
Z	original_scaffold_1674_obj_pilon	50,807
Z	original_scaffold_2242_obj_pilon	13,126
Z	original_scaffold_512_obj_pilon	17,222
Z	Super-Scaffold_29_pilon:::fragment_10:::debris	10,000
Z	original_scaffold_462_obj_pilon	11,778
Z	Super-Scaffold_29_pilon:::fragment_14:::debris	30,000
Z	original_scaffold_1741_obj_pilon	10,647
Z	Super-Scaffold_84_pilon:::fragment_6:::debris	50,000
Z	Super-Scaffold_66_pilon:::fragment_2:::debris	50,000
Z	Super-Scaffold_27_pilon:::fragment_2:::debris	50,000
Z	original_scaffold_1602_obj_pilon	10,177
Z	original_scaffold_1079_obj_pilon	7,689
Z	Super-Scaffold_66_pilon:::fragment_4:::debris	50,000
Z	Super-Scaffold_50_pilon:::fragment_2:::debris	10,000

Supplementary Table 15. Sequencing depth for each individual and their chromosomes in 162 geese.

ID	Sex	Hic_19	Hic_20	Hic_21	Hic_22	Hic_23	Hic_24	Hic_25	Hic_26	Hic_27	Hic_28	Hic_29	Hic_30	Hic_31	Hic_32	Hic_33	Hic_34	Hic_35	Hic_36	Hic_37	Hic_38	Z	W
FCG10	female	10.97	11.89	12.11	15.26	12.78	11.69	10.72	10.90	11.24	12.11	10.95	13.86	11.53	17.28	7.89	8.12	9.28	6.43	8.81	35.71	4.99	10.29
FCG12	female	11.68	12.85	12.98	16.82	13.71	12.50	11.39	11.85	12.11	13.04	11.55	14.22	12.35	18.29	9.37	8.51	9.96	6.88	9.47	37.65	5.41	11.00
FCG13	female	10.58	10.69	11.56	13.03	11.21	10.38	9.54	9.54	10.13	9.75	7.97	8.78	5.81	13.38	5.03	4.07	6.22	3.51	5.68	21.12	4.87	7.63
FCG14	female	12.41	13.17	14.33	18.26	14.54	13.16	12.08	12.39	12.55	13.59	12.26	15.32	11.98	19.70	9.46	9.25	10.19	7.13	9.73	37.39	5.70	11.11
FCG16	female	13.24	13.94	15.11	19.67	15.59	13.99	12.79	13.27	13.54	14.36	12.81	16.02	13.20	18.63	12.13	9.61	10.80	7.62	10.35	39.92	6.19	11.85
FCG17	female	10.78	11.24	12.33	15.63	12.27	11.09	10.12	10.36	10.75	11.14	9.67	12.42	9.04	14.84	7.20	6.19	8.30	5.45	7.44	28.26	4.87	9.03
FCG18	female	11.96	13.71	13.52	17.97	14.67	13.17	11.99	12.50	12.63	13.81	12.46	16.41	13.55	20.21	10.11	9.64	10.68	7.43	10.44	37.47	4.65	11.21
FCG19	female	9.44	9.99	10.51	13.36	11.03	10.02	9.12	9.50	9.73	10.55	9.33	11.88	9.72	15.31	7.17	7.10	8.42	5.70	7.75	30.90	4.31	8.76
FCG1	female	11.12	11.65	12.42	15.96	13.11	11.81	10.71	11.17	11.42	12.20	10.71	14.23	11.54	17.53	9.10	8.24	10.31	7.08	8.68	32.80	5.23	10.26
FCG20	female	11.39	12.21	12.96	17.01	13.52	12.14	11.05	11.62	11.62	12.54	11.23	13.94	11.71	17.68	9.08	8.55	9.43	6.46	9.00	32.01	5.24	10.29
FCG21	female	10.98	11.88	12.14	15.74	12.94	11.72	10.56	11.05	11.27	12.24	10.68	13.92	11.36	17.14	9.36	8.03	9.45	6.40	9.06	31.92	5.10	10.14
FCG24	female	12.85	13.68	14.74	19.21	15.35	13.93	12.59	13.16	13.24	14.34	12.82	16.29	13.69	20.77	10.50	9.83	11.14	7.67	10.85	40.31	5.85	11.97
FCG25	female	11.85	12.07	13.03	14.65	12.89	11.74	10.97	10.51	11.29	10.66	8.84	10.24	5.83	11.24	4.29	3.86	6.41	3.83	5.60	23.79	4.64	8.25
FCG26	female	12.79	13.78	14.45	18.54	15.05	13.52	12.36	12.82	13.13	14.15	12.93	16.17	13.20	20.98	9.57	10.09	10.65	7.65	10.16	35.41	5.85	11.34
FCG27	female	14.20	14.89	15.69	19.75	16.60	15.06	13.60	13.97	14.71	15.85	13.80	17.06	14.88	23.56	12.08	10.56	12.60	9.29	11.61	44.52	6.70	13.01
FCG28	female	16.36	17.68	18.98	24.34	19.42	17.40	15.87	16.36	16.87	18.28	16.18	20.78	17.12	26.29	12.29	11.90	13.29	9.98	13.27	53.03	4.65	14.65
FCG29	female	13.49	13.85	14.82	17.41	14.93	13.72	12.75	12.66	12.96	12.92	10.95	12.55	8.16	16.85	7.15	5.24	8.36	5.50	7.95	34.44	7.13	10.26
FCG2	female	12.41	13.53	13.92	17.49	14.51	13.12	11.94	12.37	12.65	13.49	12.23	16.34	13.01	22.73	9.82	8.76	10.07	7.10	9.83	36.26	4.75	11.17
FCG30	female	12.08	12.95	13.24	17.19	13.98	12.71	11.70	12.25	12.45	13.29	11.93	14.82	12.18	18.63	8.61	8.70	9.86	6.99	9.47	37.30	4.74	10.99
FCG32	female	12.71	13.92	14.67	19.40	15.05	13.67	12.32	12.73	12.99	14.11	12.73	15.77	13.24	20.20	9.24	9.47	12.00	8.61	10.35	38.89	4.11	11.63
FCG33	female	13.19	14.60	14.64	18.88	15.69	14.19	12.98	13.49	13.91	14.89	13.37	17.26	14.02	19.39	10.34	10.21	12.12	8.90	10.89	39.82	4.75	12.14
FCG34	female	12.67	13.71	13.79	17.38	14.71	13.42	12.33	12.65	13.00	14.00	12.53	15.75	12.99	20.77	9.28	8.80	10.13	6.99	9.97	35.28	4.24	11.27
FCG35	female	11.90	12.67	13.27	16.74	14.05	12.86	11.70	12.06	12.27	13.28	11.97	15.42	12.29	18.76	7.57	8.14	9.40	6.56	8.90	37.60	6.15	10.94
FCG37	female	13.74	14.95	15.58	19.94	16.31	14.74	13.32	13.82	14.26	15.43	13.83	16.89	14.34	21.37	10.14	10.34	11.60	8.33	11.30	43.07	4.42	12.20
FCG38	female	14.53	16.78	16.31	23.25	19.01	17.60	15.99	17.26	16.62	19.55	19.51	23.92	23.90	29.14	17.01	16.38	18.76	15.50	17.20	54.59	5.10	14.88
FCG39	female	11.84	12.54	12.79	15.48	13.07	12.07	11.07	11.14	11.61	11.81	10.45	12.73	9.23	15.58	7.37	6.89	9.32	6.38	7.99	31.74	3.89	9.38
FCG3	female	13.68	14.80	14.81	18.55	15.88	14.56	13.18	13.71	14.02	15.14	13.35	16.78	14.29	19.83	11.18	9.78	11.57	8.38	10.59	36.85	5.06	12.29
FCG40	female	11.18	11.86	12.10	15.13	12.96	11.90	10.81	11.28	11.55	12.56	11.43	15.11	11.87	17.71	8.64	8.74	9.75	6.82	9.25	32.11	5.88	10.21
FCG41	female	11.92	12.76	13.46	17.02	13.93	12.54	11.45	11.88	12.13	13.00	11.45	14.51	11.96	20.33	9.05	8.89	10.90	7.61	9.65	38.42	6.37	10.89
FCG42	female	11.62	12.09	13.28	16.92	13.73	12.33	11.17	11.52	11.95	12.58	11.28	14.70	11.14	18.72	9.31	7.59	9.77	6.63	9.33	39.08	4.75	10.65
FCG43	female	13.23	14.03	14.75	17.58	14.61	13.95	12.67	12.99	13.29	13.77	12.66	14.32	10.48	21.20	9.89	8.49	10.95	7.66	10.39	35.39	4.16	11.33
FCG44	female	13.66	14.99	15.44	19.86	16.13	14.67	13.26	13.82	14.15	15.27	13.78	16.63	13.99	23.95	10.58	10.94	12.85	9.08	10.66	39.02	5.24	12.38
FCG45	female	9.82	10.39	11.10	14.40	11.56	10.30	9.43	9.75	10.08	10.76	9.58	12.59	10.18	15.41	8.31	7.56	8.72	5.79	8.00	27.24	6.24	8.88
FCG47	female	7.84	8.06	8.70	11.12	9.13	8.24	7.48	7.77	7.96	8.55	7.67	9.96	8.19	12.32	5.89	5.72	6.94	4.58	6.17	21.95	3.56	7.24
FCG48	female	11.81	12.54	12.95	16.79	13.84	12.48	11.32	11.73	12.18	13.02	11.22	15.17	12.13	20.38	9.51	7.91	9.50	6.52	9.43	34.75	7.21	10.78
FCG51	female	10.85	11.65	12.29	16.19	13.09	11.74	10.71	11.05	11.25	12.19	10.77	14.16	11.56	16.81	9.27	8.31	9.32	6.37	8.97	30.57	4.69	9.84
FCG5	female	11.86	12.45	13.50	17.43	14.05	12.44	11.23	11.73	12.10	12.88	11.11	15.20	11.87	21.18	9.10	8.65	9.86	6.51	9.00	37.44	4.62	11.05
FCG6	female	12.03	12.85	13.81	17.45	14.16	12.79	11.56	11.99	12.25	13.19	11.47	15.70	12.18	20.51	8.83	8.48	10.00	6.93	9.58	36.48	5.00	10.99
FCG7	female	10.07	10.57	11.11	14.34	11.91	10.76	9.70	10.11	10.38	11.09	9.85	12.70	10.62	15.74	7.57	7.15	8.92	5.70	7.78	29.45	4.89	9.39
FCG8	female	12.21	13.31	13.71	18.44	14.86	12.88	11.44	11.99	12.87	13.90	11.69	16.68	15.41	19.58	9.68	9.66	10.51	7.61	10.02	38.26	5.07	11.82
FCG9	female	10.06	10.87	11.54	14.80	11.77	10.65	9.70	10.01	10.18	10.96	9.65	12.03	9.72	15.40	8.01	7.28	8.77	5.94	7.82	30.92	4.40	9.06
GFW11	female	14.57	15.04	16.32	20.58	16.94	15.35	13.87	14.34	14.81	15.72	13.86	17.50	14.30	22.88	11.10	10.16	11.85	8.41	11.54	43.15	4.85	13.08
GFW14	female	14.89	15.77	16.10	19.59	17.09	15.49	13.88	14.48	15.07	15.78	13.52	17.38	13.28	25.16	10.77	10.82	12.52	9.03	10.98	39.32	5.67	13.46
GFW15	female	11.62	12.23	13.11	16.55	13.54	12.09	10.86	11.34	11.79	12.45	10.82	13.94	10.88	17.75	8.92	8.33	10.25	7.16	9.29	31.47	5.29	10.74
GFW16	female	14.70	15.17	15.82	18.99	16.55	15.23	13.66	14.15	14.77	15.64	13.50	16.71	13.79	22.64	11.05	10.51	11.86	8.16	11.26	40.23	4.15	13.46
GFW17	female	12.47	13.08	13.19	15.83	13.97	13.14	11.73	12.34	12.62	13.19	11.59	14.67	11.27	17.19	9.20	9.17	10.61	7.10	9.65	36.18	5.01	11.34
GFW19	female	16.30	17.05	18.20	22.71	18.71	16.77	15.29	15.90	16.39	17.38	14.87	19.44	15.40	25.78	13.86	11.71	13.59	9.61	12.78	50.66	4.94	15.03
GFW21	female	11.73	12.38	13.30	16.11	13.17	12.32	11.06	11.51	11.56	12.10	10.71	12.28	8.67	17.78	7.97	7.52	10.50	7.11	8.50	27.46	4.13	10.17
GFW23	female	9.65	10.98	10.20	14.36	13.95	10.88	8.77	9.25	11.21	12.26	9.18	14.99	17.87	15.08	8.60	8.78	10.01	6.96	8.41	37.35	5.06	11.38
GFW24	female	13.75	14.95	15.02	18.77	15.99	14.93	13.24	13.90	14.12	15.06	13.57	16.36	13.96	24.05	11.04	10.67	12.19	8.66	11.54	38.55	7.44	12.37
GFW27	female	11.35	12.14	12.84	15.99	13.10	11.76	10.63	11.18	11.41	12.13	10.48	13.69	10.50	19.22	8.70	8.54	10.78	7.50	8.74	32.10	4.55	10.49
GFW29	female	12.55	13.06	13.66	16.51	13.98	12.67	11.49	11.90	12.49	13.13	11.12	14.75	10.79	20.57	8.65	7.99	10.79	7.50	8.95	36.02	4.09	11.50
GFW2	female	14.79	16.27	16.87	22.04	17.99	15.74	13.85	14.34	15.42	16.67	13.95	18.55	17.05	22.94	12.89	11.58	13.73	10.10	12.58	48.06	5.36	14.26
GFW30	female	10.54	10.78	11.80	14.77	12.03	10.83	9.73	10.09	10.51	11.01	9.46	12.18	9.62	16.19	7.61	7.01	8.98	5.69	7.99	30.90	5.96	9.72
GFW33	female	11.96	12.88	13.33	17.37	14.23	12.53	11.29	11.65	12.34	13.22	11.10	15.18	13.26	18.29	9.72	9.64	10.67	7.42	9.58	35.21	6.01	11.36
GFW35	female	13.55	14.45	15.04	18.95	15.88	14.09	12.74	13.23	13.89	14.74	12.34	16.03	13.49	19.79	10.54	9.87	11.77	8.33	10.51	39.41	5.64	12.29
GFW36	female	13.95	14.81	15.43	19.19	15.87	14.47	13.19	13.72	14.08	14.96	13.20	17.09	13.76	22.53	10.08	10.23	11.82	8.32	10.94	43.90	6.27	13.12

Supplementary Table 15. Sequencing depth statistics of each chromosome in 162 geese (Continued).

ID	Sex	Hic_19	Hic_20	Hic_21	Hic_22	Hic_23	Hic_24	Hic_25	Hic_26	Hic_27	Hic_28	Hic_29	Hic_30	Hic_31	Hic_32	Hic_33	Hic_34	Hic_35	Hic_36	Hic_37	Hic_38	Z	W
GFW39	female	12.69	12.83	13.98	15.94	13.55	12.53	11.64	11.70	11.95	11.79	9.94	11.68	6.77	16.57	5.52	4.93	7.51	4.72	6.68	26.32	6.25	9.62
GFW45	female	14.59	14.78	16.45	20.50	16.57	14.94	13.29	13.85	14.49	15.15	12.64	17.06	13.20	22.94	10.61	9.61	11.39	7.69	10.53	40.56	7.02	13.37
GFW46	female	12.23	12.38	13.59	16.42	13.64	12.45	11.22	11.49	11.93	12.23	10.30	13.22	9.07	17.33	7.76	6.62	8.63	5.57	8.00	30.62	5.79	10.21
GFW47	female	14.24	15.30	16.02	19.57	16.46	14.81	13.11	13.59	14.37	15.27	12.95	16.34	12.93	22.06	10.49	10.00	12.17	8.16	10.46	38.84	6.20	13.47
GFW5	female	14.11	15.03	15.67	19.24	16.12	14.54	13.23	13.79	14.29	14.94	13.02	15.84	12.84	23.25	10.05	10.12	11.89	7.89	10.53	43.04	5.77	12.90
GFW7	female	15.80	16.83	17.49	21.35	18.20	16.57	14.97	15.45	16.09	16.81	15.01	18.91	14.75	25.24	11.64	11.78	13.06	9.20	12.18	45.22	6.17	14.65
GFW8	female	11.94	12.35	13.46	17.08	13.84	12.57	11.27	11.70	12.06	12.80	11.13	14.46	11.47	18.60	9.29	8.57	10.69	7.21	9.36	35.55	5.22	10.84
GFW9	female	16.03	16.77	17.96	22.27	18.59	16.81	15.06	15.60	16.25	17.20	14.99	18.95	15.46	25.67	12.59	11.43	13.89	9.96	12.31	45.67	5.48	15.15
LHW10	female	13.68	14.72	15.12	18.53	15.60	14.05	12.78	13.14	13.74	14.38	12.42	15.48	11.95	18.36	10.16	9.35	11.71	8.26	10.30	38.31	4.33	12.83
LHW11	female	14.00	15.16	16.86	21.57	16.69	14.68	13.07	13.36	14.18	14.81	12.67	15.32	12.34	19.39	9.07	9.17	12.10	8.47	10.04	38.34	4.38	13.03
LHW1	female	12.33	12.50	14.10	16.86	13.64	12.53	11.46	11.84	12.11	12.43	10.58	12.40	8.31	13.81	7.58	5.91	9.40	6.26	8.07	33.29	6.26	10.53
LHW2	female	14.61	15.45	16.17	18.89	16.01	15.08	13.85	14.19	14.40	14.79	13.11	14.52	10.25	19.27	9.27	7.90	10.18	7.28	9.99	37.47	6.24	11.69
LHW3	female	13.31	14.20	15.17	18.56	14.83	13.71	12.67	12.87	13.25	13.45	12.04	14.69	9.79	20.73	7.79	7.25	9.60	6.38	8.64	32.84	3.29	11.29
LHW5	female	15.93	17.50	18.15	23.19	18.99	16.96	15.21	15.78	16.32	17.30	15.03	18.73	15.45	25.83	12.27	11.35	13.44	9.79	12.33	42.89	5.64	14.83
LHW6	female	8.75	9.45	9.69	11.77	9.81	8.86	7.92	8.23	8.83	9.18	7.59	10.17	7.52	12.80	6.06	5.67	7.20	4.56	6.29	24.50	4.48	8.08
LHW7	female	10.06	10.72	11.75	14.93	11.72	10.33	9.34	9.78	10.23	10.63	8.94	11.78	8.94	14.50	7.31	7.21	9.38	6.15	7.72	27.59	5.26	9.34
LHW8	female	13.51	14.57	15.50	19.42	15.79	14.05	12.60	13.12	13.70	14.33	12.18	15.65	12.12	20.86	9.89	9.07	12.17	9.02	9.98	36.47	6.06	12.67
LHW9	female	13.23	14.23	14.74	17.87	15.01	13.53	12.16	12.78	13.35	13.86	11.61	15.69	11.87	19.15	9.85	9.01	10.42	7.20	9.87	38.02	5.80	12.41
LXW10	female	13.30	13.80	14.44	16.99	14.85	13.90	12.72	12.82	13.07	13.62	11.83	14.36	9.87	17.11	8.33	7.73	9.83	6.55	9.23	32.77	5.56	10.30
LXW11	female	10.06	11.07	11.32	13.52	11.54	10.42	9.25	9.68	10.26	10.59	9.03	11.33	8.64	13.51	7.41	6.86	8.55	5.57	7.55	28.23	5.35	9.50
LXW12	female	10.65	11.78	12.25	15.23	12.41	11.03	9.92	10.38	10.81	11.48	9.74	12.64	9.81	17.30	8.14	7.51	9.70	6.68	8.23	30.69	4.07	10.09
LXW14	female	13.49	14.25	14.82	18.18	15.37	13.84	12.24	12.81	13.55	14.35	11.70	14.85	11.79	19.63	9.67	8.93	11.70	8.19	10.08	41.20	5.94	12.52
LXW16	female	12.74	13.56	13.63	16.85	14.63	13.35	12.02	12.50	12.80	13.78	12.18	16.05	12.45	19.35	9.81	9.00	10.17	7.12	10.00	37.77	3.80	11.71
LXW20	female	12.99	13.37	15.31	19.29	15.31	13.51	12.08	12.56	13.09	14.05	11.92	15.70	12.27	19.67	10.15	9.05	11.22	7.59	10.32	33.54	4.81	11.49
LXW22	female	11.00	11.24	12.01	13.32	11.76	10.58	9.94	9.59	10.40	9.80	7.85	8.81	5.31	10.86	4.18	3.55	6.14	3.56	6.22	20.52	6.88	7.29
LXW23	female	10.77	11.68	12.79	16.40	12.84	11.32	10.15	10.58	11.05	11.55	10.07	13.21	9.82	18.42	9.11	7.82	9.77	6.74	8.45	32.41	5.04	10.15
LXW24	female	14.86	15.21	15.89	17.43	15.64	14.43	13.32	13.05	14.08	13.46	10.78	12.70	7.65	17.35	6.86	5.55	8.29	5.27	7.52	33.09	6.40	10.60
LXW25	female	11.26	11.74	12.51	15.30	12.88	11.68	10.44	10.89	11.33	12.01	10.34	13.33	10.02	17.15	7.73	7.36	10.37	7.55	8.69	32.87	4.08	10.41
LXW27	female	11.68	12.60	12.72	15.65	13.28	11.97	10.76	11.26	11.66	12.13	10.66	13.15	10.58	16.35	8.90	7.87	10.11	6.66	8.96	34.05	5.41	10.45
LXW29	female	14.48	15.44	17.10	21.19	16.72	14.92	13.31	13.84	14.60	15.09	12.63	16.03	12.34	22.73	10.16	9.47	11.78	8.23	10.57	41.39	5.14	13.30
LXW2	female	15.07	15.98	17.21	21.23	17.22	15.61	14.12	14.64	15.33	15.76	13.77	17.88	12.45	21.66	10.84	10.10	12.22	8.56	11.50	40.82	4.92	13.60
LXW31	female	14.56	15.17	16.46	19.55	16.17	15.05	13.78	14.36	14.53	14.97	13.27	14.71	10.88	22.38	9.80	8.60	11.59	7.68	10.93	38.18	4.43	12.53
LXW32	female	13.97	15.00	16.08	19.86	15.79	14.56	13.24	13.72	13.84	14.50	12.91	15.64	11.40	20.09	9.77	8.08	11.74	7.86	10.37	38.73	5.07	12.50
LXW34	female	13.71	14.49	15.04	19.25	16.05	14.52	13.15	13.65	14.18	15.40	13.63	17.21	14.51	21.16	10.86	10.90	12.65	8.89	11.38	39.32	5.57	12.35
LXW36	female	11.15	11.95	12.23	15.76	12.99	11.96	10.88	11.36	11.44	12.62	11.41	14.49	11.64	15.42	8.71	8.47	11.01	8.15	9.13	32.66	5.78	10.06
LXW37	female	13.18	14.73	14.29	17.98	15.26	13.97	12.78	13.17	13.53	14.78	13.47	17.73	13.82	21.11	10.79	10.30	12.63	9.03	11.10	41.56	4.23	12.03
LXW3	female	13.26	15.09	15.53	20.05	16.31	14.50	13.05	13.69	13.89	15.39	13.60	17.65	14.49	22.50	11.15	10.52	12.54	9.27	11.44	41.71	4.40	12.88
LXW40	female	11.70	12.93	13.00	17.35	14.43	12.56	11.40	11.82	12.42	13.57	12.10	16.17	14.59	16.25	10.32	10.37	12.14	8.47	10.55	37.28	6.11	11.42
LXW42	female	17.29	18.81	19.84	25.21	20.21	18.27	16.65	17.41	17.64	18.85	16.41	20.76	16.48	26.19	13.82	12.29	15.38	10.99	13.76	49.63	4.30	16.04
LXW43	female	14.55	15.49	16.33	20.61	17.05	15.46	14.08	14.67	14.78	16.20	13.90	17.42	14.11	21.78	10.37	10.18	13.36	9.62	11.10	38.63	5.30	13.24
LXW44	female	17.68	18.39	20.09	24.10	19.70	18.56	17.23	17.82	17.59	18.73	17.20	18.19	13.25	25.21	13.28	11.61	14.71	10.77	13.41	42.63	4.00	14.29
LXW46	female	13.07	14.02	14.85	18.71	15.43	13.89	12.61	13.08	13.42	14.41	12.62	15.82	12.60	20.45	9.26	9.00	12.18	8.74	10.08	34.42	4.66	11.80
LXW48	female	13.99	14.96	16.31	21.54	16.80	14.96	13.49	14.12	14.36	15.70	13.97	17.73	14.41	21.61	11.95	10.67	13.60	9.81	11.67	39.95	5.75	12.34
LXW5	female	13.14	13.83	14.61	18.16	15.30	13.70	12.22	12.69	13.41	14.24	12.09	16.30	13.38	19.69	9.88	10.05	11.54	7.78	10.54	38.90	4.62	12.00
LXW7	female	13.21	14.19	16.85	22.48	16.25	13.98	12.84	13.49	13.49	14.52	13.14	16.87	13.18	22.91	10.03	10.17	12.41	8.55	10.68	40.14	4.54	12.78
LXW8	female	11.86	12.69	14.73	19.62	14.38	12.50	11.44	11.94	12.08	12.89	11.67	14.86	11.61	20.06	8.83	8.99	10.99	7.49	9.43	34.77	4.25	11.23
FCG11	male	11.98	12.87	13.60	17.58	14.16	12.61	11.64	11.99	12.40	13.21	11.59	15.20	12.09	19.26	8.76	8.43	9.99	7.04	9.28	31.87	10.80	6.32
FCG15	male	9.67	10.51	10.47	13.05	11.22	10.08	9.26	9.41	9.75	10.50	9.13	11.98	9.72	17.44	6.90	6.86	8.08	5.45	7.57	25.62	8.74	5.06
FCG22	male	13.03	14.05	15.03	20.02	15.65	13.85	12.75	13.09	13.60	14.54	12.87	16.16	13.39	19.95	9.66	9.47	11.28	8.15	10.50	35.94	11.68	6.87
FCG23	male	11.04	12.10	12.03	14.84	12.79	11.55	10.62	11.02	11.26	12.15	10.87	13.62	11.35	16.75	8.28	8.13	9.42	6.43	8.43	26.48	9.78	5.87
FCG31	male	10.41	11.22	11.66	15.11	12.32	10.99	10.05	10.46	10.81	11.63	10.32	13.32	11.01	16.01	7.79	7.76	8.95	6.21	8.35	27.72	9.78	5.52
FCG36	male	12.95	14.04	14.76	19.66	15.65	13.45	12.19	12.83	13.34	14.42	12.41	17.29	15.07	20.30	9.36	10.02	10.95	7.84	10.18	35.64	9.97	6.74
FCG46	male	11.32	12.15	12.86	16.88	13.40	11.93	11.03	11.43	11.60	12.64	11.36	13.96	11.61	17.03	8.13	8.82	9.67	6.78	8.59	28.91	11.02	6.04
FCG49	male	11.82	12.96	13.40	17.42	14.09	12.54	11.58	11.95	12.24	13.17	12.05	14.78	12.51	19.33	9.96	9.39	10.37	7.36	9.73	29.82	8.58	6.21
FCG4	male	11.43	12.57	12.60	16.30	13.57	12.20	11.21	11.67	11.90	12.72	11.29	14.53	12.19	19.62	9.20	8.36	9.88	6.99	9.44	30.51	11.47	6.11
FCG50	male	13.42	13.44	14.46	16.99	14.65	13.21	12.15	12.36	13.11	12.97	10.84	12.54	9.01	17.98	9.03	6.56	8.81	5.43	8.57	28.36	9.99	6.62
GFW10	male	13.26	13.71	14.99	19.16	16.38	13.83	12.23	12.73	13.93	15.23	12.29	17.67	16.56	21.48	10.81	9.88	11.46	8.38	10.56	36.32	7.58	6.92
GFW12	male	14.41	15.62	16.18	20.64	16.66	14.90	13.65	14.24	14.69	15.67	13.65	17.61	13.99	23.52	11.41	10.55	12.92	9.67	11.41	36.31	9.85	7.38

Supplementary Table 15. Sequencing depth statistics of each chromosome in 162 geese (Continued).

ID	Sex	Hic_19	Hic_20	Hic_21	Hic_22	Hic_23	Hic_24	Hic_25	Hic_26	Hic_27	Hic_28	Hic_29	Hic_30	Hic_31	Hic_32	Hic_33	Hic_34	Hic_35	Hic_36	Hic_37	Hic_38	Z	W
GFW13	male	13.57	14.20	14.95	18.46	15.74	14.09	12.79	13.26	13.64	14.63	12.46	16.18	12.52	21.08	9.51	9.47	11.49	8.26	10.87	38.33	9.55	7.08
GFW18	male	13.40	13.90	14.70	18.12	15.09	13.81	12.48	12.90	13.41	14.14	12.26	15.88	12.75	21.60	10.16	9.49	11.07	7.78	10.19	31.45	10.28	6.77
GFW1	male	14.58	16.08	16.22	20.54	17.07	15.53	14.13	14.50	14.79	15.95	14.06	18.05	14.68	22.50	11.28	10.65	11.63	8.32	10.92	34.55	13.08	7.42
GFW20	male	12.56	13.49	14.30	17.89	14.33	12.75	11.62	12.14	12.56	13.24	11.21	14.73	11.55	20.03	9.06	8.52	9.93	6.81	9.34	31.17	12.45	6.43
GFW22	male	12.75	13.52	14.50	18.76	14.83	13.11	12.02	12.32	12.86	13.71	11.78	15.23	12.55	20.19	9.83	8.90	10.74	7.21	10.20	32.35	10.34	6.59
GFW25	male	12.67	13.56	13.85	17.30	14.71	13.41	12.29	12.70	12.93	14.06	12.67	16.47	13.03	22.67	10.24	10.61	11.50	8.14	10.28	32.56	9.05	6.73
GFW26	male	14.12	14.78	15.90	19.78	16.26	14.37	13.11	13.49	14.23	14.91	12.88	16.96	13.36	19.86	9.89	10.18	12.04	8.32	10.74	34.14	10.12	7.06
GFW28	male	12.27	13.09	13.85	17.13	13.98	12.54	11.41	11.77	12.45	12.96	10.90	13.58	10.88	19.77	8.92	8.22	10.04	6.64	9.00	28.72	8.94	6.31
GFW31	male	13.82	14.38	15.97	20.02	15.94	13.93	12.58	12.96	13.77	14.30	11.84	15.03	11.99	21.37	9.81	8.66	10.75	6.97	10.23	34.28	11.40	7.04
GFW32	male	11.25	12.23	12.57	15.56	12.97	11.38	10.42	10.90	11.41	11.85	10.10	13.78	10.89	18.07	8.51	8.44	9.69	6.49	8.80	33.58	8.67	5.89
GFW34	male	13.04	13.90	14.51	18.13	15.13	13.76	12.47	12.89	13.35	14.19	12.69	15.63	12.27	20.10	10.16	9.61	10.72	7.48	10.39	32.79	7.07	6.89
GFW37	male	11.63	12.40	12.88	16.32	13.39	12.10	11.04	11.36	11.80	12.58	10.90	14.25	11.39	19.74	8.40	9.07	10.81	7.49	8.85	30.10	14.25	6.13
GFW38	male	16.18	17.40	18.47	23.75	18.79	16.73	15.38	15.90	16.47	17.43	15.11	19.65	15.63	24.87	13.07	11.89	14.39	10.64	12.90	42.87	7.17	8.21
GFW3	male	12.86	13.39	14.00	17.05	14.50	13.08	12.04	12.47	12.95	13.66	11.70	14.46	12.00	19.83	9.61	9.03	10.42	7.21	9.78	26.72	10.74	6.59
GFW40	male	14.90	15.85	16.81	21.10	17.64	15.54	14.03	14.56	15.42	16.09	13.94	17.75	14.48	23.76	11.37	10.97	12.51	8.61	11.72	39.08	11.55	7.75
GFW41	male	12.19	12.96	13.89	17.56	14.26	12.58	11.48	11.96	12.45	13.07	10.96	14.36	11.32	22.06	9.80	8.69	11.12	7.56	9.63	33.68	8.46	6.42
GFW42	male	11.68	12.29	13.23	16.40	13.50	11.99	10.82	11.20	11.63	12.32	10.68	13.06	10.93	19.90	8.92	8.20	9.65	6.53	8.85	29.69	7.98	6.06
GFW43	male	14.26	15.47	16.14	20.25	16.38	14.85	13.66	14.14	14.45	15.49	13.45	16.87	13.65	24.35	11.06	10.38	12.09	8.61	11.02	35.30	9.05	7.41
GFW44	male	13.14	13.88	14.75	18.67	15.78	13.89	12.48	13.01	13.45	14.34	12.52	15.33	12.52	21.42	10.77	9.82	11.97	8.34	10.74	34.64	9.21	7.11
GFW48	male	11.98	12.64	13.52	16.70	13.93	12.32	11.14	11.50	12.16	12.66	11.07	14.24	11.31	19.49	8.61	8.33	9.58	6.59	9.09	32.79	9.60	6.35
GFW49	male	12.94	13.51	15.07	19.56	15.10	13.33	12.13	12.60	12.95	13.62	11.98	14.79	12.51	19.32	9.54	9.26	11.23	7.51	10.11	32.17	10.11	6.71
GFW4	male	13.65	14.61	15.38	19.03	15.77	14.19	12.81	13.15	13.90	14.62	12.40	16.41	12.83	23.02	10.61	9.60	11.10	7.68	10.42	32.83	7.50	6.96
GFW50	male	13.48	14.21	14.91	18.77	15.68	14.12	12.89	13.36	13.83	14.83	13.21	16.41	13.19	21.75	11.27	10.38	12.01	8.60	10.71	32.78	8.77	6.92
GFW6	male	15.60	16.91	17.82	22.19	18.18	16.07	14.60	15.08	15.70	16.48	13.97	17.16	14.38	25.08	11.15	11.06	13.01	9.45	11.38	40.32	10.51	7.91
LHW4	male	11.42	12.57	12.93	16.42	13.57	12.13	11.04	11.42	11.84	12.36	10.94	13.44	10.58	17.90	8.63	7.72	9.66	6.65	8.72	25.84	9.19	5.85
LXW13	male	10.59	11.35	11.60	14.28	12.10	10.71	9.82	10.29	10.81	11.11	9.68	12.40	9.47	17.24	7.71	6.97	9.62	6.42	8.10	26.49	12.35	5.52
LXW15	male	12.26	13.15	13.80	17.62	14.43	12.82	11.51	12.03	12.45	13.34	11.34	14.24	11.57	17.25	9.50	9.37	10.95	7.24	9.47	30.62	11.77	6.44
LXW17	male	13.57	14.68	15.08	18.97	16.17	14.09	12.58	13.28	13.97	14.82	12.67	16.90	14.50	19.09	10.25	9.52	11.36	7.79	10.52	36.74	11.96	7.21
LXW18	male	14.17	15.39	16.86	22.82	18.05	14.63	13.16	13.65	15.03	16.22	13.14	17.54	18.28	21.03	12.09	11.22	13.24	8.94	11.65	38.26	10.17	7.61
LXW19	male	14.26	15.24	15.55	18.95	16.18	14.75	13.53	14.02	14.52	15.15	13.36	16.42	12.42	22.98	10.73	9.42	12.13	8.52	11.21	34.86	11.69	7.17
LXW1	male	12.03	12.60	13.68	16.78	13.80	12.39	11.38	11.58	11.95	12.39	10.49	13.10	9.90	13.99	7.70	7.03	8.54	5.72	8.40	27.04	10.32	5.98
LXW21	male	10.84	11.62	12.60	16.68	12.93	11.35	10.42	10.82	11.15	12.02	10.77	14.10	10.83	16.23	8.73	7.74	10.21	7.11	8.74	26.03	10.78	5.76
LXW26	male	13.13	14.55	15.16	19.28	15.53	13.53	12.11	12.68	13.53	14.15	11.81	15.68	13.20	21.39	9.85	9.59	11.45	7.51	10.30	35.72	8.65	6.74
LXW28	male	12.84	13.46	15.27	18.90	14.88	12.96	11.78	12.14	12.89	13.09	10.97	13.59	10.09	18.08	8.46	7.86	10.15	6.84	8.91	32.53	10.48	6.42
LXW30	male	11.00	11.38	12.37	15.10	12.58	11.11	10.02	10.34	11.06	11.29	9.80	12.50	9.29	16.93	8.57	7.27	9.35	6.14	8.25	28.33	9.95	5.73
LXW33	male	11.54	12.57	13.61	17.59	14.07	12.02	10.88	11.21	12.11	12.71	11.07	15.01	12.44	19.68	9.09	8.78	10.58	7.29	9.39	31.55	6.88	6.23
LXW35	male	11.98	13.07	13.97	18.55	14.28	12.53	11.61	12.09	12.27	13.38	12.08	15.20	12.61	22.52	9.88	9.59	11.97	8.61	10.02	32.75	10.96	6.39
LXW38	male	12.66	13.46	13.87	17.50	14.79	13.43	12.20	12.71	12.92	14.07	12.84	16.03	12.84	19.07	10.90	9.61	11.96	8.51	10.60	33.61	9.35	6.64
LXW39	male	12.93	13.88	14.55	18.55	15.15	13.77	12.51	13.05	13.33	14.30	13.00	15.22	12.77	19.18	10.41	10.27	12.46	9.00	10.48	34.17	8.15	6.68
LXW41	male	14.27	15.55	15.98	19.16	15.87	14.71	13.69	14.22	14.21	15.19	13.48	15.29	11.56	19.55	10.65	9.03	12.60	8.74	11.15	35.18	11.36	7.05
LXW45	male	14.75	15.52	16.40	20.35	17.28	15.67	14.19	14.71	15.21	16.55	14.50	17.72	14.45	20.39	11.36	10.47	13.58	9.79	12.03	37.79	8.56	7.45
LXW47	male	12.31	13.43	14.22	18.17	14.36	12.97	12.03	12.53	12.53	13.56	12.19	14.85	11.53	17.43	10.47	8.42	11.84	8.52	10.07	33.21	13.26	6.36
LXW49	male	12.53	13.43	14.52	18.54	14.41	13.15	12.10	12.59	12.73	13.50	12.21	15.12	11.14	16.60	9.08	8.82	12.06	8.45	9.74	29.45	12.98	6.39
LXW4	male	13.75	15.05	15.39	18.67	15.82	14.17	12.89	13.43	13.99	14.76	12.59	16.08	12.87	21.04	9.77	10.54	11.80	7.85	10.66	33.43	10.24	7.11
LXW50	male	13.74	14.88	14.82	18.70	15.89	14.53	13.32	13.77	14.16	15.35	13.76	17.01	14.95	21.21	11.36	10.93	13.31	9.79	11.28	37.14	11.74	7.32
LXW6	male	13.58	14.50	15.21	19.14	15.54	14.00	12.91	13.38	13.77	14.64	12.83	16.16	12.90	20.57	10.45	9.03	12.13	8.77	10.69	34.72	9.26	7.01
LXW9	male	15.24	15.95	17.14	21.00	17.13	15.32	13.98	14.34	15.10	15.60	13.14	16.30	12.81	21.18	9.67	8.59	11.15	7.49	10.46	36.79	9.74	7.68

Supplementary Table 16. Genome assemblies used for gene families and phylogenetic analysis.

Common name	Species	Genus	Family	Assembly
Xingguo gray goose	*Anser cygnoides*	*Anser*	*Anatidae*	This study
Tianfu goose[47]	*Anser cygnoides*	*Anser*	*Anatidae*	GCA_013030995.1
Zhedong white goose[48]	*Anser cygnoides*	*Anser*	*Anatidae*	GCF_000971095.1
Sichuan white goose[49]	*Anser cygnoides*	*Anser*	*Anatidae*	GCA_002166845.1
Mallard[63]	*Anas platyrhynchos*	*Anas*	*Anatidae*	GCA_008746955.1
Eastern spot-billed duck	*Anas zonorhyncha*	*Anas*	*Anatidae*	GCA_002224875.1
Pekin duck[40]	*Anas platyrhynchos*	*Anas*	*Anatidae*	GCA_003850225.1
Tufted duck	*Aythya fuligula*	*Aythya*	*Anatidae*	GCF_009819795.1
Muscovy duck	*Cairina moschata*	*Cairina*	*Anatidae*	GCA_009194515.1
Ruddy duck	*Oxyura jamaicensis*	*Oxyura*	*Anatidae*	GCF_011077185.1
Pink-footed goose[51]	*Anser brachyrhynchus*	*Anser*	*Anatidae*	GCA_002592135.1
Black swan	*Cygnus atratus*	*Cygnus*	*Anatidae*	GCF_013377495.1
Magpie goose[64]	*Anseranas semipalmata*	*Anseranas*	*Anatidae*	GCA_013399115.1
Turkey[53]	*Meleagris gallopavo*	*Meleagris*	*Phasianidae*	GCF_000146605.3
Chicken[52]	*Gallus gallus*	*Gallus*	*Phasianidae*	GCF_000002315.6

Note: Shaed species are used for phylogenetic analysis.

Supplementary Table 17. GO analysis for the lineage-specific gene families in XGG compared to TFG, SCW and ZDW.

GO term	Category	Function	*p*-value
GO:0005869	GO Cellular Components	dynactin complex	3.53E-13
GO:0005884	GO Cellular Components	actin filament	1.28E-10
GO:0030137	GO Cellular Components	COPI-coated vesicle	8.59E-07
GO:0005925	GO Cellular Components	focal adhesion	7.09E-06
GO:0005938	GO Cellular Components	cell cortex	1.09E-03

Supplementary Table 18. GO analysis for the significant expansion gene families in XGG.

GO term	Category	Function	*p*-value
GO:0005005	GO Molecular Functions	transmembrane-ephrin receptor activity	1.80E-16
GO:0035637	GO Biological Processes	multicellular organismal signaling	4.67E-09
GO:0016309	GO Molecular Functions	1-phosphatidylinositol-5-phosphate 4-kinase activity	2.09E-07
GO:0030424	GO Cellular Components	axon	3.47E-07
GO:1902495	GO Cellular Components	transmembrane transporter complex	5.85E-07
GO:0090625	GO Biological Processes	mRNA cleavage involved in gene silencing by siRNA	8.30E-07
GO:0044420	GO Cellular Components	extracellular matrix component	9.37E-07
GO:0046718	GO Biological Processes	viral entry into host cell	1.58E-06
GO:0044449	GO Cellular Components	contractile fiber part	8.58E-06
GO:0045322	GO Molecular Functions	unmethylated CpG binding	1.14E-05
GO:0000139	GO Cellular Components	Golgi membrane	1.54E-05
GO:0042541	GO Biological Processes	hemoglobin biosynthetic process	2.43E-05
GO:0030900	GO Biological Processes	forebrain development	2.53E-05
GO:0016594	GO Molecular Functions	glycine binding	7.23E-05
GO:0046982	GO Molecular Functions	protein heterodimerization activity	1.41E-04
GO:0016607	GO Cellular Components	nuclear speck	1.57E-04
GO:0007157	GO Biological Processes	heterophilic cell-cell adhesion via plasma membrane cell adhesion molecules	1.78E-04
GO:0030246	GO Molecular Functions	carbohydrate binding	2.12E-04
GO:0001525	GO Biological Processes	angiogenesis	3.20E-04
GO:0031589	GO Biological Processes	cell-substrate adhesion	3.28E-04
GO:0005509	GO Molecular Functions	calcium ion binding	3.63E-04
GO:0006907	GO Biological Processes	pinocytosis	3.85E-04
GO:0007628	GO Biological Processes	adult walking behavior	5.49E-04
GO:0002230	GO Biological Processes	positive regulation of defense response to virus by host	6.80E-04
GO:1904019	GO Biological Processes	epithelial cell apoptotic process	7.80E-04
GO:0005930	GO Cellular Components	axoneme	8.72E-04
GO:0009611	GO Biological Processes	response to wounding	1.16E-03
GO:0097190	GO Biological Processes	apoptotic signaling pathway	1.17E-03

Supplementary Table 18. GO analysis for the significant expansion gene families in XGG (Continued).

GO term	Category	Function	*p*-value
GO:0120035	GO Biological Processes	regulation of plasma membrane bounded cell projection organization	1.37E-03
GO:0001540	GO Molecular Functions	amyloid-beta binding	1.42E-03
GO:0032386	GO Biological Processes	regulation of intracellular transport	1.45E-03
GO:0019932	GO Biological Processes	second-messenger-mediated signaling	1.74E-03
GO:0005912	GO Cellular Components	adherens junction	1.89E-03
GO:0042063	GO Biological Processes	gliogenesis	2.54E-03
GO:0006303	GO Biological Processes	double-strand break repair via nonhomologous end joining	3.07E-03
GO:1990778	GO Biological Processes	protein localization to cell periphery	4.03E-03
GO:0098900	GO Biological Processes	regulation of action potential	4.82E-03
GO:0030155	GO Biological Processes	regulation of cell adhesion	4.91E-03
GO:0009991	GO Biological Processes	response to extracellular stimulus	5.80E-03
GO:0031646	GO Biological Processes	positive regulation of neurological system process	6.95E-03

Supplementary Table 19. Overlapping genes identified by zFst between white and gray geese and zHp in white geese.

Chromosome	zFst	zHp	Gene_Start	Gene_End	Gene_Name
15	11.23	-3.84	15,758,873	15,772,560	*EDNRB2*
15	11.23	-3.84	15,773,733	15,779,279	*POLR1D*
2	10.05	-3.40	18,809,517	18,832,692	*EIF3E*
38	6.72	-3.44	38,080,915	38,141,883	*AGTPBP1*
38	6.39	-3.62	66,751,352	66,789,274	*CSPG4*
2	5.98	-3.73	18,858,633	18,911,400	*RSPO2*
38	5.74	-4.13	66,642,732	66,664,363	*SNX18*
6	5.59	-3.66	17,455,295	17,593,698	*TTC7B*
5	5.08	-3.46	32,077,378	32,166,339	*PTPN13*
38	4.86	-4.24	23,725,129	23,779,550	*ZNF462*
38	4.51	-4.21	24165,321	24,181,476	*TMEM38B-B*
3	4.48	-3.44	67,976,648	68,050,654	*SYNE1*
19	3.63	-3.24	5,397,937	5,422,453	*TOM1L1*

Supplementary Table 20. Frequency of the 14-bp insertion in wild, white, and gray goose populations.

Breeds (plumage color)	Total number	Genotype (Genotype frequency)		
		+/+	+/wt	wt/wt
Anser albifrons (gray)*	6	0(0)	0(0)	6(1)
Anser anser (gray)*	6	0(0)	0(0)	6(1)
Anser cygnoides (gray)*	59	0(0)	0(0)	59(1)
Anser erythropus (gray)*	6	0(0)	0(0)	6(1)
Anser fabalis (gray)*	6	0(0)	0(0)	6(1)
Cygnus columbianus (white)*	6	0(0)	0(0)	6(1)
Fengcheng gray goose (gray)	51	0(0)	4(0.08)	47(0.92)
Shitou goose (gray)	100	0(0)	0(0)	100(1)
Wuzong goose (gray)	100	0(0)	0(0)	100(1)
Xingguo gray goose (gray)	60	0(0)	0(0)	60(1)
Landaise goose (gray)	50	0(0)	0(0)	50(1)
Guangfeng white goose (white)	50	50(1)	0(0)	0(0)
Huoyan goose (white)	60	60(1)	0(0)	0(0)
Lianhua white goose (white)	11	11(1)	0(0)	0(0)
Linxian white goose (white)	50	50(1)	0(0)	0(0)
Mingbei white goose (white)	51	51(1)	0(0)	0(0)

Note: * represents wild population; + represents 14-bp insertion and wt represents wild-type.

Supplementary Table 21. The variants with extremely significant allele frequencies in wild, white, and gray geese.

CHR	Position	Alternative allele	Reference allele	Gene	description	Cluster	MAF	MAC	Number of alleles
15	15763328	G	A	*EDNRB2*	intronic	Anser	0.2349	39	166
						Cygnus	1	12	12
						Gray	0.0429	31	722
						White	1	444	444
15	15763384	G	C	*EDNRB2*	intronic	Anser	0.3976	66	166
						Cygnus	0	0	12
						Gray	0.0055	4	722
						White	1	444	444
15	15764237	G	C	*EDNRB2*	intronic	Anser	0.1867	31	166
						Cygnus	0	0	12
						Gray	0.0152	11	722
						White	1	444	444
15	15764499	A	G	*EDNRB2*	intronic	Anser	0.0783	13	166
						Cygnus	1	12	12
						Gray	0.0055	4	722
						White	1	444	444
15	15764637	GCACAGGT GAGCTCT	G	*EDNRB2*	Frameshift insertion, exon3:c.602_603insCA CAGGTGAGCTCT:p.S 201fs	Anser	0	0	166
						Cygnus	0	0	12
						Gray	0.0055	4	722
						White	1	444	444
15	15765937	C	A	*EDNRB2*	intronic	Anser	0.0301	5	166
						Cygnus	0	0	12
						Gray	0.0055	4	722
						White	0.9932	441	444
15	15765943	G	A	*EDNRB2*	intronic	Anser	0.1325	22	166
						Cygnus	0.5	6	12
						Gray	0.0402	29	722
						White	0.9932	441	444
15	15765978	T	G	*EDNRB2*	intronic	Anser	0.0301	5	166
						Cygnus	0	0	12
						Gray	0.0402	29	722
						White	0.9932	441	444
15	15765994	T	C	*EDNRB2*	intronic	Anser	0.0422	7	166
						Cygnus	0	0	12
						Gray	0.0402	29	722
						White	0.9932	441	444
15	15766491	C	T	*EDNRB2*	intronic	Anser	0.3735	62	166
						Cygnus	0	0	12
						Gray	0.0152	11	722
						White	0.991	440	444
15	15766502	T	C	*EDNRB2*	intronic	Anser	0.3253	54	166

						Cygnus	0	0	12
						Gray	0.0055	4	722
						White	0.9887	439	444
15	15767006	A	G	*EDNRB2*	intronic	Anser	0.0241	4	166
						Cygnus	0	0	12
						Gray	0.0083	6	722
						White	0.964	428	444
15	15767117	A	G	*EDNRB2*	intronic	Anser	0.0783	13	166
						Cygnus	0	0	12
						Gray	0.0055	4	722
						White	0.9865	438	444
15	15767847	G	A	*EDNRB2*	intronic	Anser	0.5241	87	166
						Cygnus	1	12	12
						Gray	0.0263	19	722
						White	0.9887	439	444
15	15769453	T	C	*EDNRB2*	UTR3, c.*850C>T	Anser	0.0301	5	166
						Cygnus	0	0	12
						Gray	0.0139	10	722
						White	0.9617	427	444
15	15769504	C	T	*EDNRB2*	UTR3, c.*901T>C	Anser	0.0301	5	166
						Cygnus	0	0	12
						Gray	0.0319	23	722
						White	0.9685	430	444
15	15769505	A	G	*EDNRB2*	UTR3, c.*902G>A	Anser	0.0301	5	166
						Cygnus	0	0	12
						Gray	0.0152	11	722
						White	0.9617	427	444
15	15769683	A	G	*EDNRB2*	UTR3, c.*1080G>A	Anser	0.0482	8	166
						Cygnus	0	0	12
						Gray	0.0125	9	722
						White	0.9595	426	444
15	15770566	T	C	*EDNRB2*	UTR3, c.*1963C>T	Anser	0.0241	4	166
						Cygnus	0	0	12
						Gray	0.0152	11	722
						White	0.9842	437	444
15	15770880	G	A	*EDNRB2*	UTR3, c.*2277A>G	Anser	0.4398	73	166
						Cygnus	1	12	12
						Gray	0.0457	33	722
						White	0.9887	439	444
15	15775255	A	G	*POLR1D*	intronic	Anser	0.0241	4	166
						Cygnus	0	0	12
						Gray	0.0485	35	722
						White	0.9865	438	444
15	15777558	T	G	*POLR1D*	intronic	Anser	0.0181	3	166
						Cygnus	0	0	12

						Gray	0.0471	34	722
						White	0.982	436	444
15	15778568	T	C	*POLR1D*	intronic	Anser	0.3795	63	166
						Cygnus	1	12	12
						Gray	0.0471	34	722
						White	0.9707	431	444
15	15778575	A	G	*POLR1D*	intronic	Anser	0.3735	62	166
						Cygnus	1	12	12
						Gray	0.0457	33	722
						White	0.9685	430	444
15	15778592	T	C	*POLR1D*	intronic	Anser	0.3133	52	166
						Cygnus	0	0	12
						Gray	0.0499	36	722
						White	0.9595	426	444
15	15778959	G	A	*POLR1D*	intronic	Anser	0.2289	38	166
						Cygnus	0	0	12
						Gray	0.0499	36	722
						White	0.9707	431	444
15	15778991	C	CCAGCG CTGCTA	*POLR1D*	intronic	Anser	0.1747	29	166
						Cygnus	0	0	12
						Gray	0.0499	36	722
						White	0.9707	431	444

Note: MAF, minor allele frequency; MAC, minor allele count in cluster; Anser, ancestral populations and closely related species.

Supplementary Table 22. Overlapping genes identified by zFst between XGG and other geese and zHp in XGG population.

Chr	zFst	zHp	Gene_start	Gene_end	Gene
1	4.2423609	-3.97783751	164365239	164387414	*SLC5A8*
2	5.1030351	-3.16406827	99128590	99194125	*PPP1R17*
6	5.3002111	-4.00008866	61246543	61396443	*PLEKHA7*
9	3.6587609	-5.16182216	17309730	17330843	*Hic_asm_9.361*
15	4.4666206	-4.32233659	16795527	16819262	*CHIC1*
15	3.7679185	-4.23615863	18373952	18475699	*AMMECR1*
15	8.361944	-3.95922	5804230	5991030	*DIAPH2*
15	4.0971025	-3.89774323	18486092	18530364	*TMEM164*
15	5.0179989	-3.15456429	16795527	16819262	*CHIC1*
15	9.3826581	-3.1264262	15758873	15772560	*EDNRB*
16	3.9087894	-3.12637206	11720364	11739084	*LUC7L*
26	4.1295195	-4.99522952	4635431	4640908	*Hic_asm_26.322*
28	4.1747604	-3.88297635	5633672	5674798	*MYO1F*
38	6.6557467	-5.05047353	60381450	60398646	*AGXT2*
38	4.8800648	-4.96661217	59045958	59138210	*CCBE1*
38	4.0933975	-4.91338601	60410068	60430582	*PRLR*
38	7.1068973	-4.382922	38833035	38854797	*FBP2*
38	6.1352576	-4.22897146	38817894	38823677	*CTSL*
38	5.4233538	-4.04068453	60573405	60625640	*SPEF2*
38	4.5662638	-3.99617173	62224295	62249090	*DAB2*
38	6.9723499	-3.17589598	9001616	9192436	*EDIL3*

Supplementary Figures

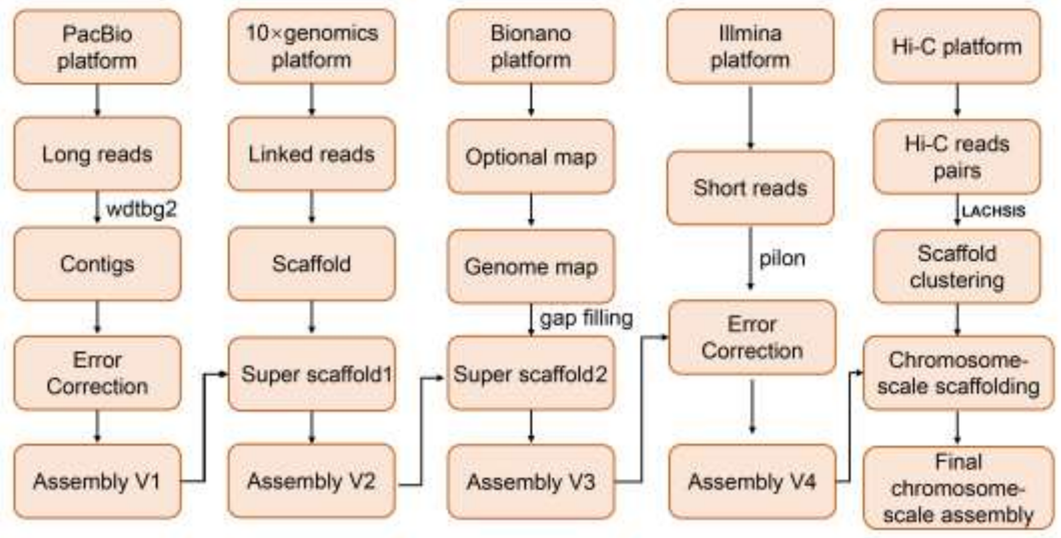

Supplementary Figure 1. The genome assembling flowchart of XGG in Novogene Company.

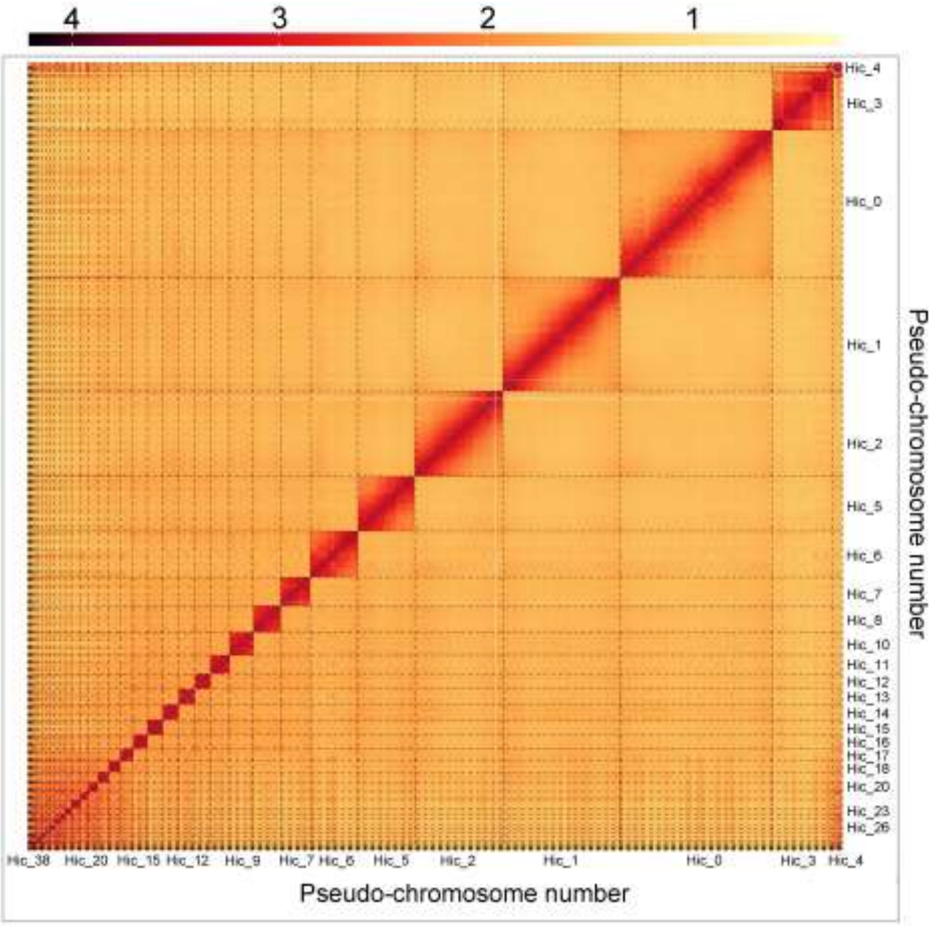

Supplementary Figure 2. The Hi-C interaction contact heatmap of goose pseudo-chromosome genome assembly.

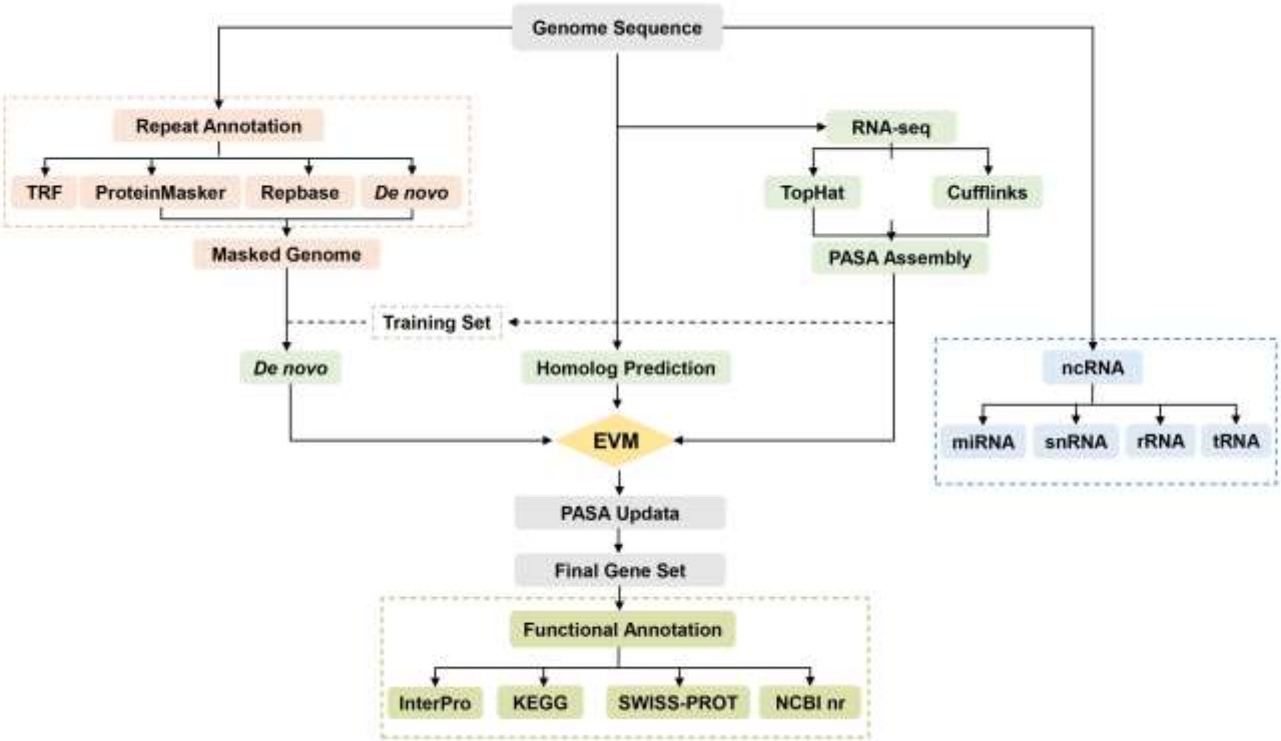

Supplementary Figure 3. The genome annotation pipeline of XGG genome in Novogene Company.

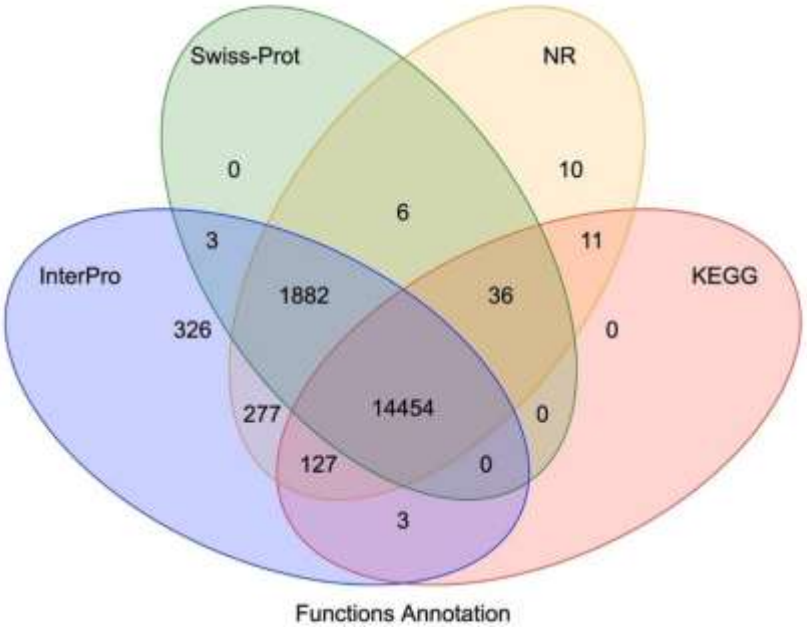

Supplementary Figure 4. Venn diagram of gene function annotation results of different protein databases.

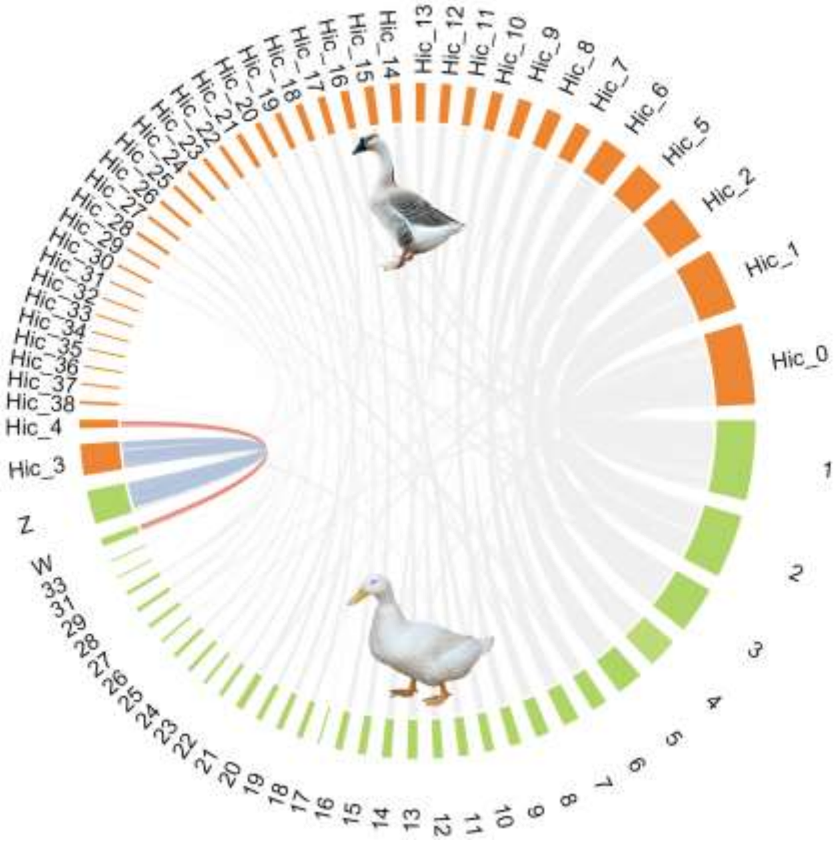

Supplementary Figure 5. The collinearity results between XGG and Pekin duck. The orange and green blocks represent the chromosomes of XGG and Pekin duck, respectively.

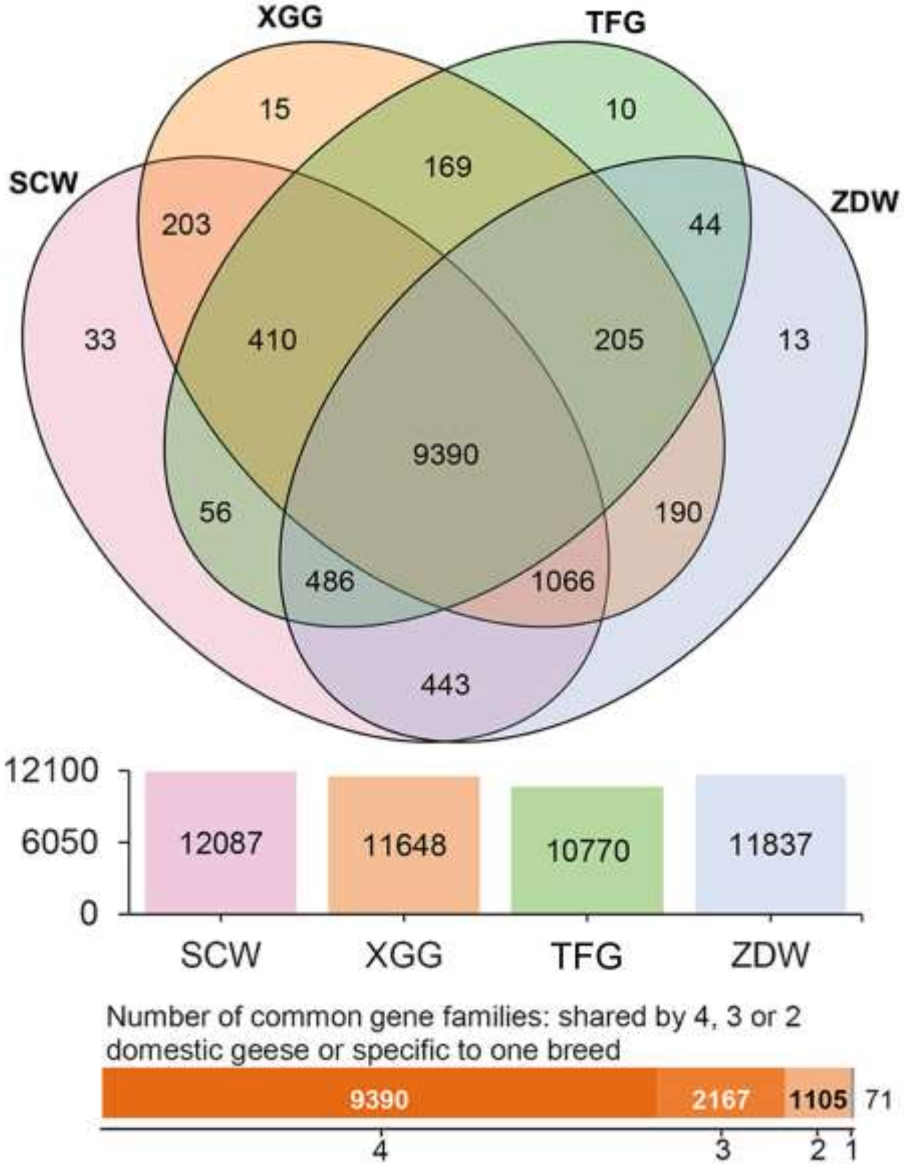

Supplementary Figure 6. The number of shared and unique gene families among four domestic geese. SCW, Sichuan white goose; XGG, Xingguo gray goose; TFG, Tianfu goose; ZDW, Zhedong white goose.

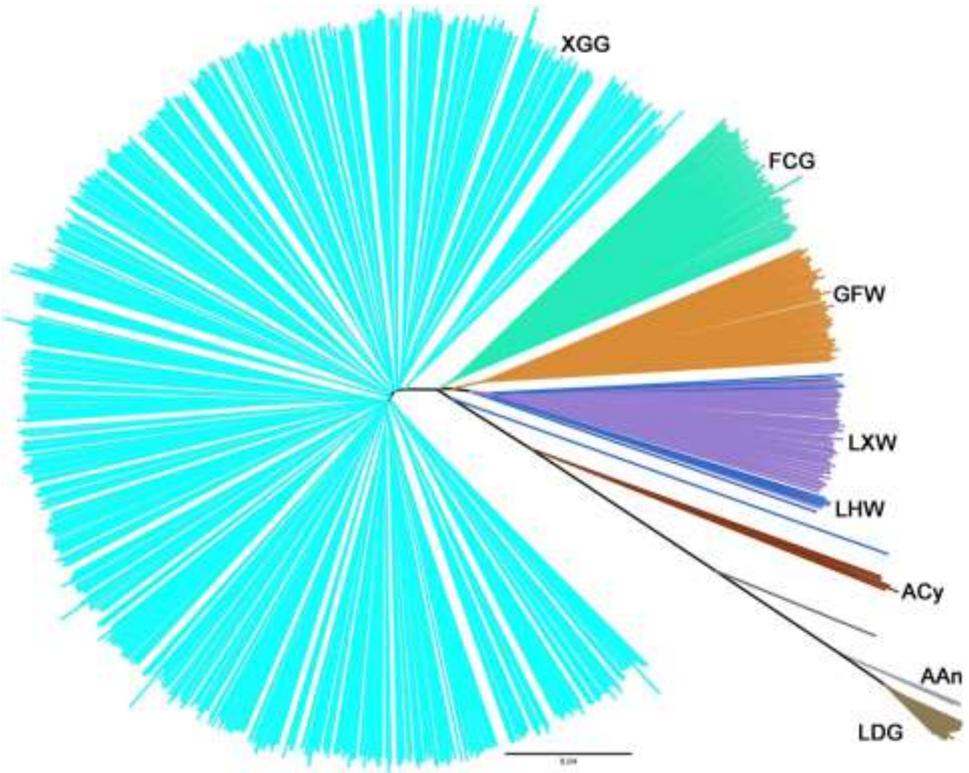

Supplementary Figure 7. Neighbor-joining tree of 845 geese. XGG, Xingguo gray goose; FCG, Fengcheng gray goose; GFW, Guangfeng white goose; LXW, Lingxian white goose; LHW, Lianhua white goose; ACy, Swan goose; AAn, Greylag goose; LDG, Landaise goose.

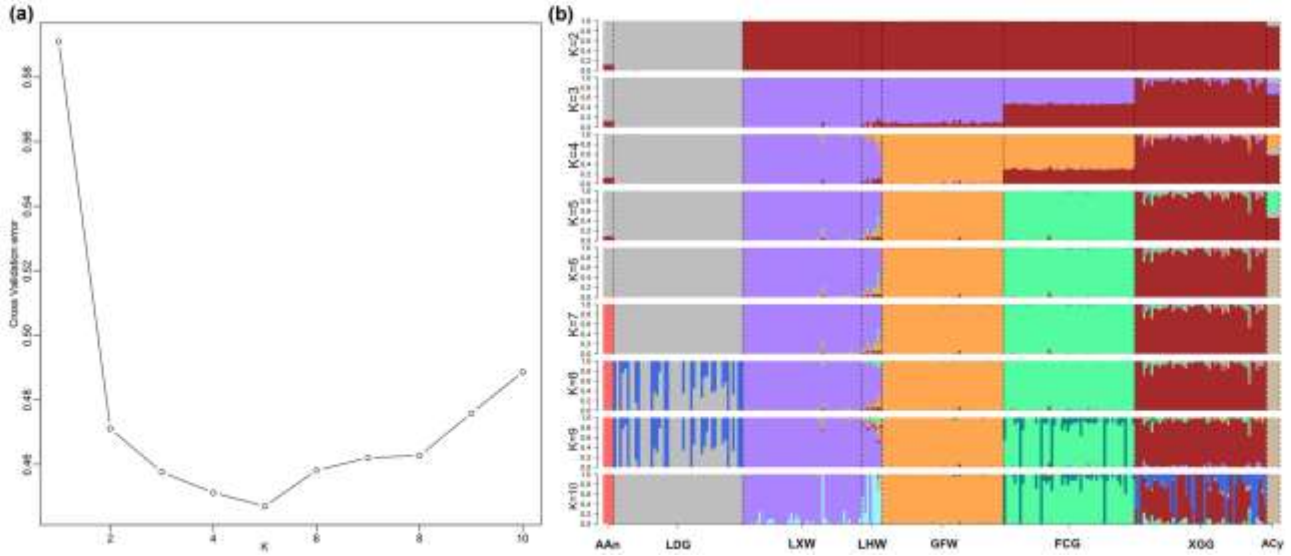

Supplementary Figure 8. Population structure analysis with the maximum likelihood. (a) Cross-validation error from K = 2 to K = 10 in ADMIXTURE analysis. (b) Population-structure plots with K = 2~10.

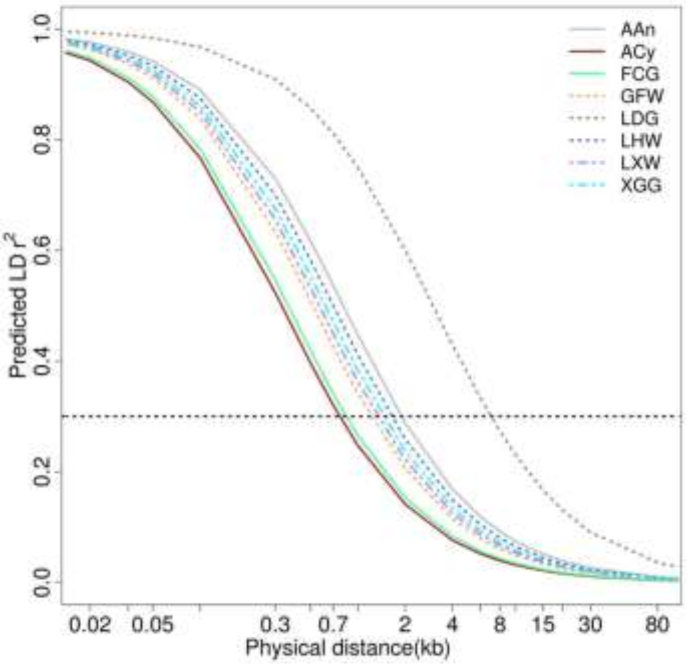

Supplementary Figure 9. Linkage disequilibrium (LD) analysis for six geese breeds and two wild species. LD values were estimated using whole-genome sequence data of all individuals for each population. The y-axis indicates the physical distance, the ordinate indicates the predicted LD(r^2) value, and the horizontal dashed line indicates the threshold line (r^2 = 0.3).

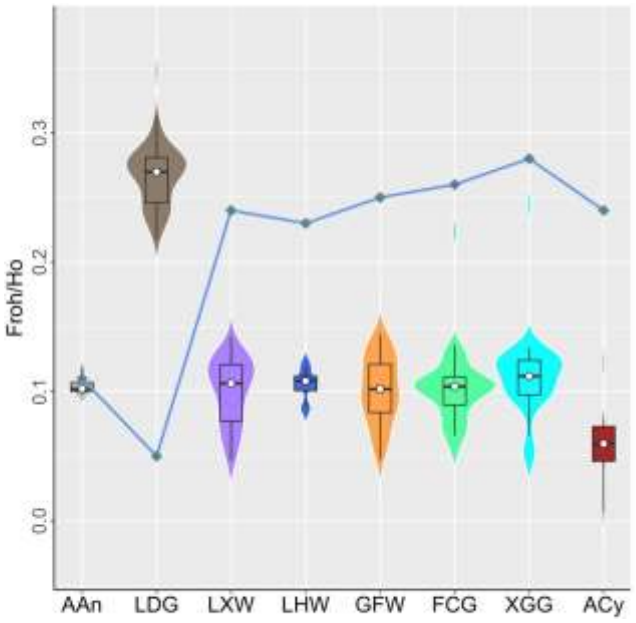

Supplementary Figure 10. The proportion of F_{ROH} and Ho for six geese breeds and two wild species. The box plot shows the F_{ROH} values of each population, and the broken line shows the Ho.

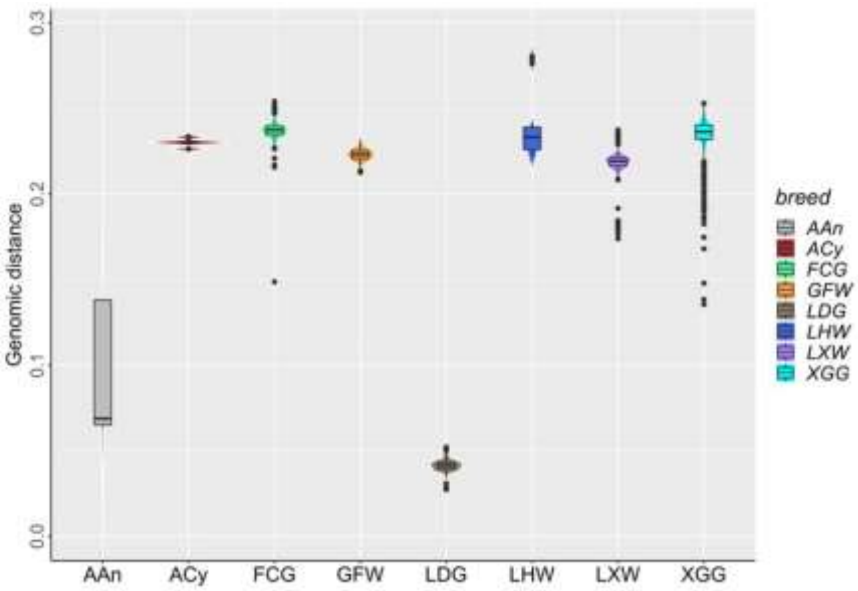

Supplementary Figure 11. The genetic distance (DST) of each population.

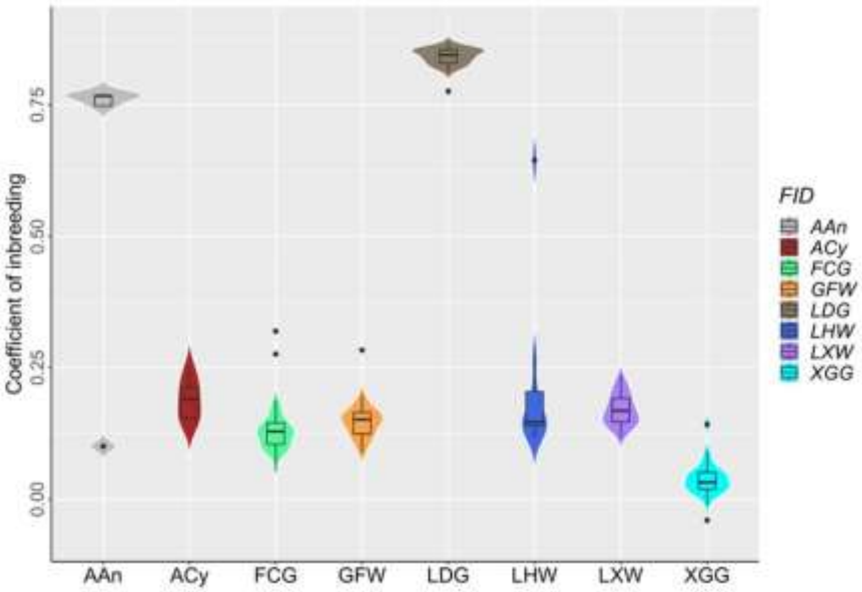

Supplementary Figure 12. The inbreeding coefficient (F) of each population.

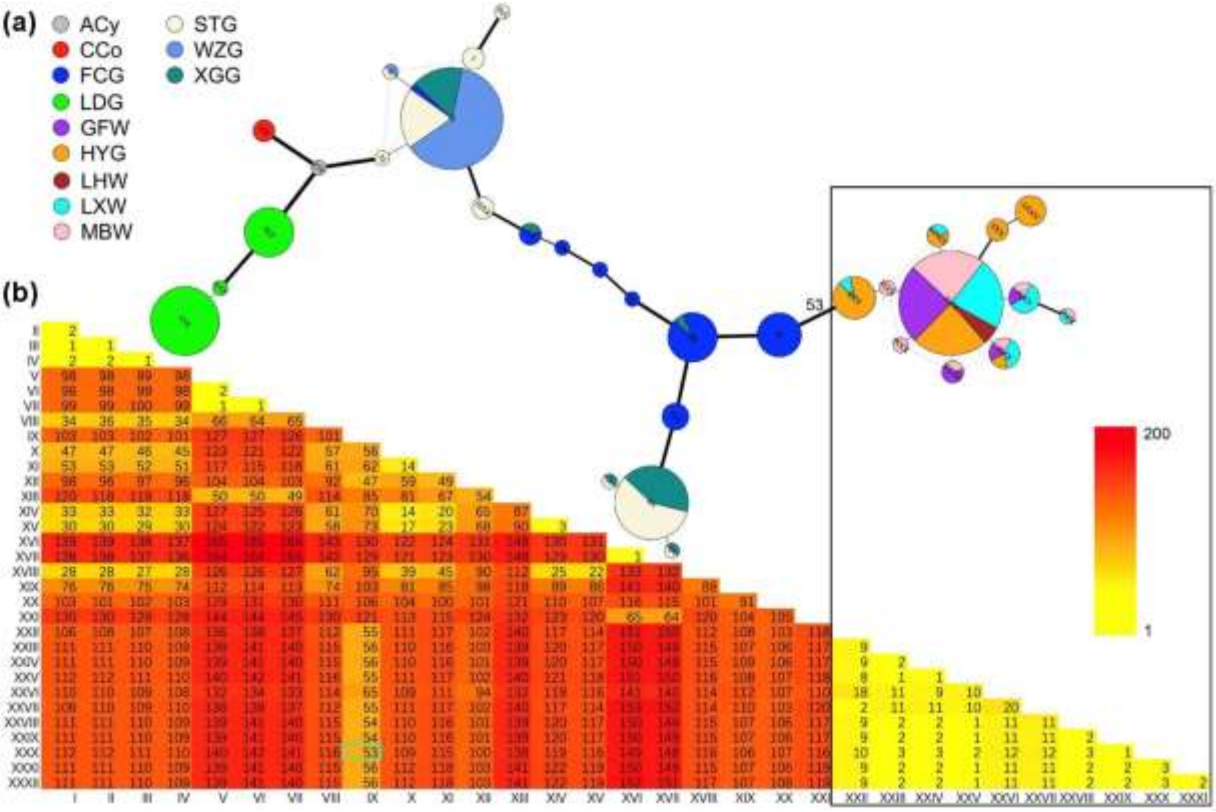

Supplementary Figure 13. Haplotype network and the difference between 32 haplotypes of 285 variants in *EDNRB2* and *POLR1D* locus. (a) The same haplotype network diagram as Fig. 4d with haplotype ID and the corresponding haplotype differences. (b) The number represents the difference between every two haplotypes, the black box represents the haplotype of white plumage geese, and the cyan box indicates the lowest haplotype difference between gray and white geese.

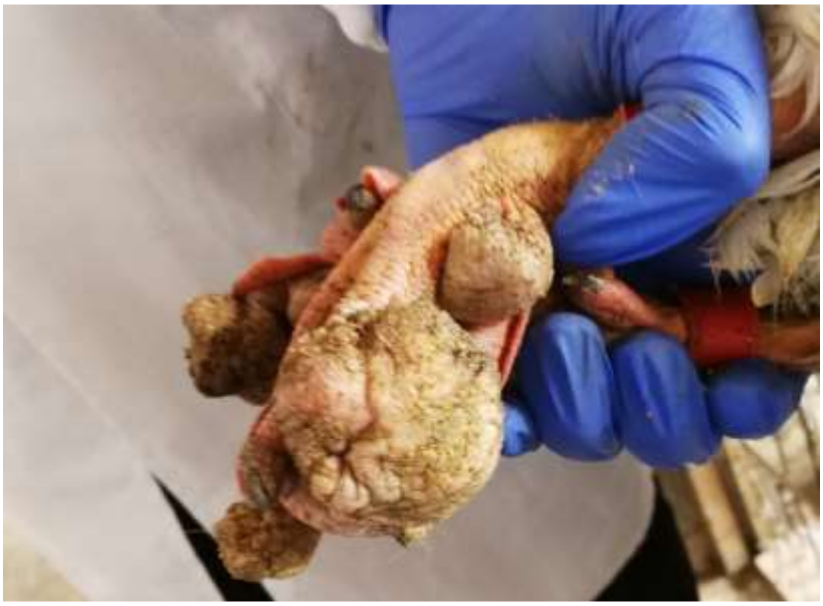

Supplementary Figure 14. Epidermal cysts occur on the feet of XGG population.

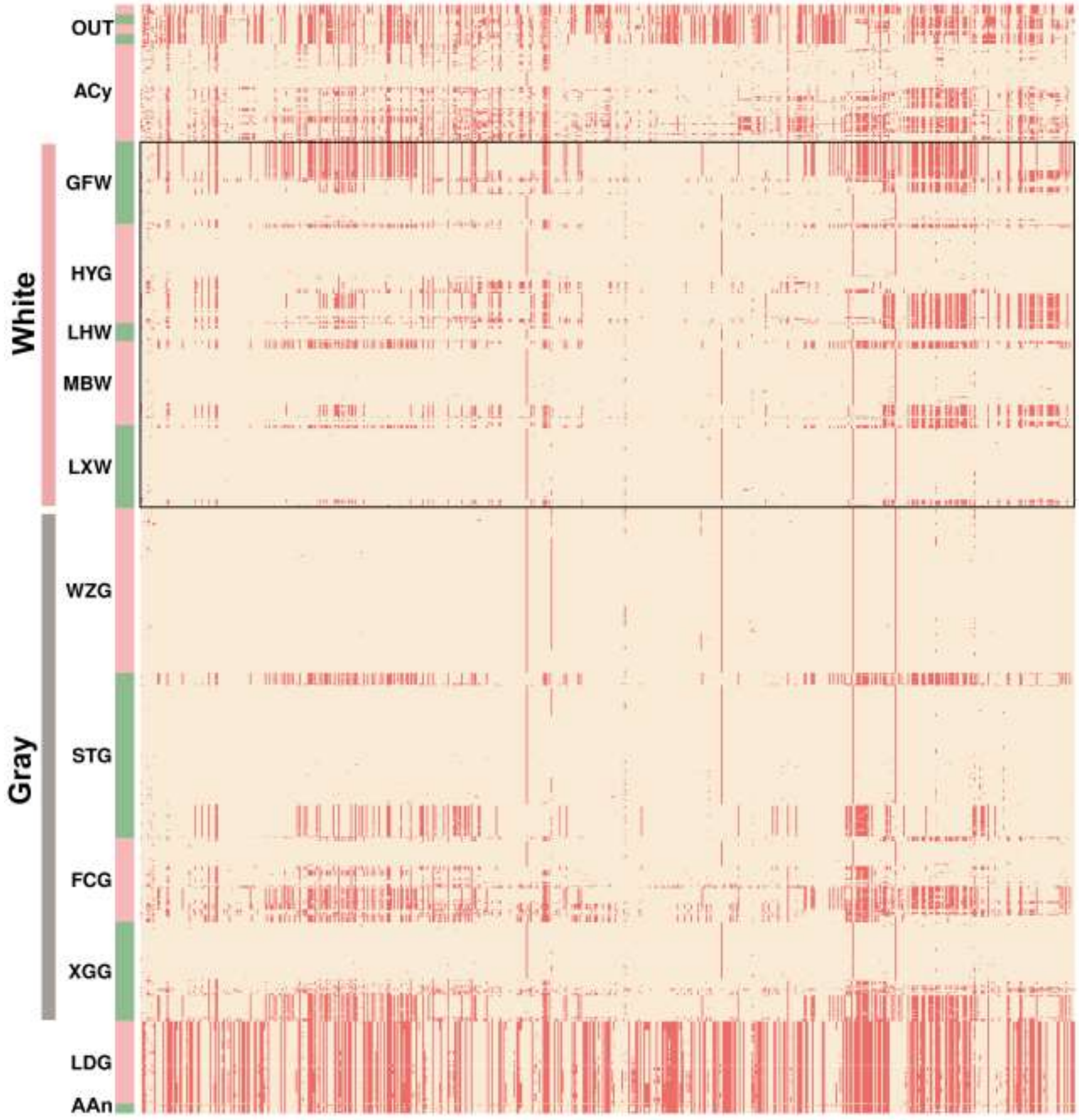

Supplementary Figure 15. Haplotype analysis of *KIT* gene. White represents the Chinese white geese, and Gray represents Chinese gray geese. The black box represents the haplotypes of the white geese population, and the beige and orange colors represent the high and low frequency alleles in the white geese, respectively.

Supplementary References

1. Ruan, J. & Li, H. Fast and accurate long-read assembly with wtdbg2. *Nat. Methods* **17**, 155-158 (2020).
2. Adey, A. et al. In vitro, long-range sequence information for de novo genome assembly via transposase contiguity. *Genome Res.* **24**, 2041-2049 (2014).
3. Shelton, J.M. et al. Tools and pipelines for BioNano data: molecule assembly pipeline and FASTA super scaffolding tool. *BMC Genomics* **16**, 734 (2015).
4. Li, H. & Durbin, R. Fast and accurate short read alignment with Burrows-Wheeler transform. *Bioinformatics* **25**, 1754-1760 (2009).
5. Walker, B.J. et al. Pilon: an integrated tool for comprehensive microbial variant detection and genome assembly improvement. *PLoS One* **9**, e112963 (2014).
6. Li, H. et al. The Sequence Alignment/Map format and SAMtools. *Bioinformatics* **25**, 2078-2079 (2009).
7. Burton, J.N. et al. Chromosome-scale scaffolding of de novo genome assemblies based on chromatin interactions. *Nat. Biotechnol.* **31**, 1119-1125 (2013).
8. Parra, G., Bradnam, K. & Korf, I. CEGMA: a pipeline to accurately annotate core genes in eukaryotic genomes. *Bioinformatics* **23**, 1061-1067 (2007).
9. Manni, M., Berkeley, M.R., Seppey, M., Simao, F.A. & Zdobnov, E.M. BUSCO Update: Novel and Streamlined Workflows along with Broader and Deeper Phylogenetic Coverage for Scoring of Eukaryotic, Prokaryotic, and Viral Genomes. *Mol. Biol. Evol.* **38**, 4647-4654 (2021).
10. Camacho, C. et al. BLAST+: architecture and applications. *BMC Bioinformatics* **10**, 421 (2009).
11. Birney, E., Clamp, M. & Durbin, R. GeneWise and Genomewise. *Genome Res.* **14**, 988-995 (2004).
12. Levy Karin, E., Mirdita, M. & Soding, J. MetaEuk-sensitive, high-throughput gene discovery, and annotation for large-scale eukaryotic metagenomics. *Microbiome* **8**, 48 (2020).
13. Meng, X. & Ji, Y. Modern Computational Techniques for the HMMER Sequence Analysis. *ISRN. Bioinform.* **2013**, 252183 (2013).
14. Benson, G. Tandem repeats finder: a program to analyze DNA sequences. *Nucleic Acids Res.* **27**, 573-580 (1999).
15. Bao, W., Kojima, K.K. & Kohany, O. Repbase Update, a database of repetitive elements in eukaryotic genomes. *Mob. DNA* **6**, 11 (2015).
16. Tarailo-Graovac, M. & Chen, N. Using RepeatMasker to identify repetitive elements in genomic sequences. *Curr. Protoc. Bioinformatics* **Chapter 4**, Unit 4.10 (2009).
17. Xu, Z. & Wang, H. LTR_FINDER: an efficient tool for the prediction of full-length LTR retrotransposons. *Nucleic Acids Res.* **35**, W265-268 (2007).
18. Price, A.L., Jones, N.C. & Pevzner, P.A. De novo identification of repeat families in large genomes. *Bioinformatics* **21 Suppl 1**, i351-358 (2005).
19. Bao, Z. & Eddy, S.R. Automated de novo identification of repeat sequence families in sequenced genomes. *Genome Res.* **12**, 1269-1276 (2002).
20. Edgar, R.C. Search and clustering orders of magnitude faster than BLAST.

Bioinformatics **26**, 2460-2461 (2010).
21. Birney, E. & Durbin, R. Using GeneWise in the Drosophila annotation experiment. *Genome Res.* **10**, 547-548 (2000).
22. Stanke, M., Steinkamp, R., Waack, S. & Morgenstern, B. AUGUSTUS: a web server for gene finding in eukaryotes. *Nucleic Acids Res.* **32**, W309-W312 (2004).
23. Parra, G., Blanco, E. & Guigo, R. GeneID in Drosophila. *Genome Res.* **10**, 511-515 (2000).
24. Burge, C. & Karlin, S. Prediction of complete gene structures in human genomic DNA. *J. Mol. Biol.* **268**, 78-94 (1997).
25. Majoros, W.H., Pertea, M. & Salzberg, S.L. TigrScan and GlimmerHMM: two open source ab initio eukaryotic gene-finders. *Bioinformatics* **20**, 2878-2879 (2004).
26. Korf, I. Gene finding in novel genomes. *BMC Bioinformatics* **5**, 59 (2004).
27. Trapnell, C., Pachter, L. & Salzberg, S.L. TopHat: discovering splice junctions with RNA-Seq. *Bioinformatics* **25**, 1105-1111 (2009).
28. Trapnell, C. et al. Transcript assembly and quantification by RNA-Seq reveals unannotated transcripts and isoform switching during cell differentiation. *Nat. Biotechnol.* **28**, 511-515 (2010).
29. Haas, B.J. et al. Automated eukaryotic gene structure annotation using EVidenceModeler and the Program to Assemble Spliced Alignments. *Genome Biol.* **9**, R7 (2008).
30. Altschul, S.F. et al. Gapped BLAST and PSI-BLAST: a new generation of protein database search programs. *Nucleic Acids Res.* **25**, 3389-3402 (1997).
31. Bairoch, A. & Apweiler, R. The SWISS-PROT protein sequence data bank and its supplement TrEMBL in 1999. *Nucleic Acids Res.* **27**, 49-54 (1999).
32. O'Leary, N.A. et al. Reference sequence (RefSeq) database at NCBI: current status, taxonomic expansion, and functional annotation. *Nucleic Acids Res.* **44**, D733-D745 (2016).
33. Finn, R.D. et al. The Pfam protein families database. *Nucleic Acids Res.* **38**, D211-D222 (2010).
34. Du, J. et al. KEGG-PATH: Kyoto encyclopedia of genes and genomes-based pathway analysis using a path analysis model. *Mol. Biosyst.* **10**, 2441-2447 (2014).
35. Apweiler, R. et al. The InterPro database, an integrated documentation resource for protein families, domains and functional sites. *Nucleic Acids Res.* **29**, 37-40 (2001).
36. Ashburner, M. et al. Gene ontology: tool for the unification of biology. The Gene Ontology Consortium. *Nat. Genet.* **25**, 25-29 (2000).
37. Lowe, T.M. & Eddy, S.R. tRNAscan-SE: a program for improved detection of transfer RNA genes in genomic sequence. *Nucleic Acids Res.* **25**, 955-964 (1997).
38. Nawrocki, E.P., Kolbe, D.L. & Eddy, S.R. Infernal 1.0: inference of RNA alignments. *Bioinformatics* **25**, 1335-1337 (2009).
39. Griffiths-Jones, S. et al. Rfam: annotating non-coding RNAs in complete genomes. *Nucleic Acids Res.* **33**, D121-D124 (2005).
40. Li, J. et al. A new duck genome reveals conserved and convergently evolved chromosome architectures of birds and mammals. *Gigascience* **10**, giaa142 (2021).
41. Chen, C. et al. TBtools: An Integrative Toolkit Developed for Interactive Analyses of Big Biological Data. *Mol. Plant.* **13**, 1194-1202 (2020).

42. Kendig, K.I. et al. Sentieon DNASeq Variant Calling Workflow Demonstrates Strong Computational Performance and Accuracy. *Front. Genet.* **10**, 736 (2019).
43. McKenna, A. et al. The Genome Analysis Toolkit: a MapReduce framework for analyzing next-generation DNA sequencing data. *Genome Res.* **20**, 1297-1303 (2010).
44. Danecek, P. & McCarthy, S.A. BCFtools/csq: haplotype-aware variant consequences. *Bioinformatics* **33**, 2037-2039 (2017).
45. Chang, C.C. et al. Second-generation PLINK: rising to the challenge of larger and richer datasets. *Gigascience* **4**, 7 (2015).
46. Davies, R.W., Flint, J., Myers, S. & Mott, R. Rapid genotype imputation from sequence without reference panels. *Nat. Genet.* **48**, 965-969 (2016).
47. Li, Y. et al. Pacific Biosciences assembly with Hi-C mapping generates an improved, chromosome-level goose genome. *Gigascience* **9**, 1-8 (2020).
48. Lu, L. et al. The goose genome sequence leads to insights into the evolution of waterfowl and susceptibility to fatty liver. *Genome Biol.* **16**, 89 (2015).
49. Gao, G. et al. Genome and metagenome analyses reveal adaptive evolution of the host and interaction with the gut microbiota in the goose. *Sci. Rep.* **6**, 32961 (2016).
50. Wang, W. et al. First de novo whole genome sequencing and assembly of the bar-headed goose. *PeerJ* **8**, e8914 (2020).
51. Pujolar, J.M., Dalen, L., Olsen, R.A., Hansen, M.M. & Madsen, J. First de novo whole genome sequencing and assembly of the pink-footed goose. *Genomics* **110**, 75-79 (2018).
52. Hillier, L.W. et al. Sequence and comparative analysis of the chicken genome provide unique perspectives on vertebrate evolution. *Nature* **432**, 695-716 (2004).
53. Dalloul, R.A. et al. Multi-platform next-generation sequencing of the domestic turkey (Meleagris gallopavo): genome assembly and analysis. *PLoS Biol.* **8**, e1000475 (2010).
54. Zhang, G.J. et al. Comparative genomics reveals insights into avian genome evolution and adaptation. *Science* **346**, 1311-1320 (2014).
55. Colquitt, B.M., Mets, D.G. & Brainard, M.S. Draft genome assembly of the Bengalese finch, Lonchura striata domestica, a model for motor skill variability and learning. *Gigascience* **7**, 1-6 (2018).
56. Rhie, A. et al. Towards complete and error-free genome assemblies of all vertebrate species. *Nature* **592**, 737-746 (2021).
57. Penalba, J.V. et al. Genome of an iconic Australian bird: High-quality assembly and linkage map of the superb fairy-wren (Malurus cyaneus). *Mol. Ecol. Resour.* **20**, 560-578 (2020).
58. Leskovec, J. & Sosic, R. SNAP: A General Purpose Network Analysis and Graph Mining Library. *ACM Trans. Intell. Syst. Technol.* **8**, 1 (2016).
59. Ghosh, S. & Chan, C.K. Analysis of RNA-Seq Data Using TopHat and Cufflinks. *Methods Mol. Biol.* **1374**, 339-361 (2016).
60. Wu, Y. et al. Population genomic data reveal genes related to important traits of quail. *Gigascience* **7**, giy049 (2018).
61. Shen, Q.K. et al. Genomic Analyses of Unveil Helmeted Guinea Fowl (Numida meleagris) Domestication in West Africa. *Genome Biol. Evol.* **13**, evab090 (2021).

62. Laine, V.N. et al. Evolutionary signals of selection on cognition from the great tit genome and methylome. *Nature Communications* **7**, 10474 (2016).
63. Zhu, F. et al. Three chromosome-level duck genome assemblies provide insights into genomic variation during domestication. *Nature Communications* **12**, 5932 (2021).
64. Armstrong, J. et al. Progressive Cactus is a multiple-genome aligner for the thousand-genome era. *Nature* **587**, 246-251 (2020).
