# Supplementary material for: Chromosome-level genome and population genomics reveal evolutionary characteristics and conservation status of Chinese indigenous geese
Source: Commun Biol. 2022 Nov 7;5:1191. doi: 10.1038/s42003-022-04125-x (PMC9640629; doi:10.1038/s42003-022-04125-x)
Supplement: Supplementary file 3 — Description of Additional Supplementary Files [file 42003_2022_4125_MOESM3_ESM.pdf]

Description of Additional Supplementary Files

File name: Supplementary Data 1
Description: Positively selected genes identified in XGG genome.

File name: Supplementary Data 2
Description: Functional categories enrichment of positively selected genes in XGG.

File name: Supplementary Data 3
Description: Sample information for whole genome resequencing.
