# Supplementary material for: Chromosome-level genome and population genomics reveal evolutionary characteristics and conservation status of Chinese indigenous geese
Source: Commun Biol. 2022 Nov 7;5:1191. doi: 10.1038/s42003-022-04125-x (PMC9640629; doi:10.1038/s42003-022-04125-x)
Supplement: Supplementary file 7 — Reporting Summary [file 42003_2022_4125_MOESM7_ESM.pdf]

nature portfolio

Corresponding author(s): Hao Chen

Last updated by author(s): Sep 3, 2022

Reporting Summary

Nature Portfolio wishes to improve the reproducibility of the work that we publish. This form provides structure for consistency and transparency in reporting. For further information on Nature Portfolio policies, see our Editorial Policies and the Editorial Policy Checklist.

Statistics

For all statistical analyses, confirm that the following items are present in the figure legend, table legend, main text, or Methods section.

n/a	Confirmed	
☒	☐	The exact sample size (n) for each experimental group/condition, given as a discrete number and unit of measurement
☒	☐	A statement on whether measurements were taken from distinct samples or whether the same sample was measured repeatedly
☐	☒	The statistical test(s) used AND whether they are one- or two-sided<br>*Only common tests should be described solely by name; describe more complex techniques in the Methods section.*
☒	☐	A description of all covariates tested
☒	☐	A description of any assumptions or corrections, such as tests of normality and adjustment for multiple comparisons
☐	☒	A full description of the statistical parameters including central tendency (e.g. means) or other basic estimates (e.g. regression coefficient) AND variation (e.g. standard deviation) or associated estimates of uncertainty (e.g. confidence intervals)
☒	☐	For null hypothesis testing, the test statistic (e.g. F, t, r) with confidence intervals, effect sizes, degrees of freedom and P value noted<br>*Give P values as exact values whenever suitable.*
☐	☒	For Bayesian analysis, information on the choice of priors and Markov chain Monte Carlo settings
☒	☐	For hierarchical and complex designs, identification of the appropriate level for tests and full reporting of outcomes
☒	☐	Estimates of effect sizes (e.g. Cohen's d, Pearson's r), indicating how they were calculated

Our web collection on statistics for biologists contains articles on many of the points above.

Software and code

Policy information about availability of computer code

Data collection	No commercial/custom code was used for data collection.
Data analysis	Software used for data analysis included wtdbg v2.4, fragScaff v140324.1, BWA v0.7.17, pilon v1.23, LACHESIS v201701, CEGMA v2.5, BUSCO v5.2.2, RepeatMasker v4.07, BLAST v2.2.28, and GeneWise v2.4.1, Augustus v.3.3.3, Geneid v1.4, Genescan v3.1.2, GlimmerHMM v3.04, SNAP v2013.11.29, LTR_FINDER v1.05, RepeatScout v1.05, RepeatModeler v1.0.3, EVidenceModeler v1.1.1, PASA v2.4.1, TopHat v2.0.8, Cufflinks v2.1.1, tRNAscan-SE v1.4, INFERNAL v1.1.2, SAMtools v1.10, TBtools v1.06, OrthoFinder v2.4.0, MAFFT v7.407, trimAl v1.4, RAxML v8.2.12, IQ-TREE v2.1.1, PAML v4.9j, Tracer v1.7.1, CAFÉ v.4.2.1, Sentieon v201711.03, GATK v4.0.12, BCFtools v1.15, STITCH v1.68, SnpEff v5.0, PLINK v1.9, PHYLIP v3.69, Figtree v1.4.2, GCTA v1.92, ADMIXTURE v1.3.0, Beagle v5.1, pegas v1.1.

For manuscripts utilizing custom algorithms or software that are central to the research but not yet described in published literature, software must be made available to editors and reviewers. We strongly encourage code deposition in a community repository (e.g. GitHub). See the Nature Portfolio guidelines for submitting code & software for further information.

Data

Policy information about availability of data

All manuscripts must include a data availability statement. This statement should provide the following information, where applicable:

- Accession codes, unique identifiers, or web links for publicly available datasets
- A description of any restrictions on data availability
- For clinical datasets or third party data, please ensure that the statement adheres to our policy

The Xingguo gray goose genome reported in this study has been deposited in the Genome Warehouse in BIG Data Center (https://bigd.big.ac.cn/gwh/) under accession number GWHBAAW00000000. The genome resequencing data for 994 domestic and wild geese have been deposited in the NCBI database as BioProject PRJNA678815. The SNP dataset of domestic and wild geese has been deposited in the Genome Variation Map in BIG Data Center (https://bigd.big.ac.cn/gvm/) under accession number GVM000131. These data are available to anyone for the purpose of reproducing or extending the analysis. Analytical pipelines and code are available on the Zenodo (https://zenodo.org/; doi:10.5281/zenodo.6613753).

Human research participants

Policy information about studies involving human research participants and Sex and Gender in Research.

Reporting on sex and gender	NA
Population characteristics	NA
Recruitment	NA
Ethics oversight	NA

Note that full information on the approval of the study protocol must also be provided in the manuscript.

Field-specific reporting

Please select the one below that is the best fit for your research. If you are not sure, read the appropriate sections before making your selection.

☒ Life sciences ☐ Behavioural & social sciences ☐ Ecological, evolutionary & environmental sciences

For a reference copy of the document with all sections, see nature.com/documents/nr-reporting-summary-flat.pdf

Life sciences study design

All studies must disclose on these points even when the disclosure is negative.

Sample size	Blood and tissue samples from an adult female Xingguo gray goose were used for De novo genome sequencing. Blood samples from 994 domestic and wild geese were used for whole genome resequencing.
Data exclusions	No data exclusion was performed.
Replication	The studied phenotypes was not available in other data sets. However, we identified associations of markers with white feather that had previously been associated with other bird studies.
Randomization	No randomization was used.
Blinding	Not relevant.

Reporting for specific materials, systems and methods

We require information from authors about some types of materials, experimental systems and methods used in many studies. Here, indicate whether each material, system or method listed is relevant to your study. If you are not sure if a list item applies to your research, read the appropriate section before selecting a response.

Materials & experimental systems

n/a	Involved in the study
☒	☐ Antibodies
☒	☐ Eukaryotic cell lines
☒	☐ Palaeontology and archaeology
☐	☒ Animals and other organisms
☒	☐ Clinical data
☒	☐ Dual use research of concern

Methods

n/a	Involved in the study
☒	☐ ChIP-seq
☒	☐ Flow cytometry
☒	☐ MRI-based neuroimaging

Animals and other research organisms

Policy information about studies involving animals; ARRIVE guidelines recommended for reporting animal research, and Sex and Gender in Research

Laboratory animals	The study did not involve laboratory animals.
Wild animals	Five swan geese (Anser cygnoides) and five greylag geese (Anser anser) were captured with a net from Poyang Lake (28°52'N, 116° 23'E) of Jiangxi, China. After the blood was taken from under the wings, hemostasis was carried out and then released on the spot.
Reporting on sex	Determination of goose sex chromosomes by sequencing depth and collinearity.
Field-collected samples	Whole blood samples were collected from adult geese in Jiangxi province, China.
Ethics oversight	All procedures used for this study and involved birds complied with guidelines for the care and utility of experimental animals established by the Ministry of Agriculture of China.

Note that full information on the approval of the study protocol must also be provided in the manuscript.
